# Exploring EEG resting state differences in autism: sparse findings from a large cohort

**DOI:** 10.1186/s13229-025-00647-3

**Published:** 2025-02-24

**Authors:** Adam J.O. Dede, Wenyi Xiao, Nemanja Vaci, Michael X. Cohen, Elizabeth Milne

**Affiliations:** 1 School of Psychology, University of Sheffield, Sheffield, S10 2TN UK; 2 Department of Medical and Social Sciences, Northwestern University, Chicago, USA; 3 Donders Institute for Brain, Cognition and Behaviour, Radboud University, Nijmegen, 6525 EN The Netherlands

**Keywords:** Autism diagnosis, Biomarkers, Big data, Heterogeneity, Neurodevelopmental disorders, Resting state, Replication, NIMH data archive

## Abstract

**Background:**

Autism is a complex neurodevelopmental condition, the precise neurobiological underpinnings of which remain elusive. Here, we focus on group differences in resting state EEG (rsEEG). Although many previous reports have pointed to differences between autistic and neurotypical participants in rsEEG, results have failed to replicate, sample sizes have typically been small, and only a small number of variables are reported in each study.

**Methods:**

Here, we combined five datasets to create a large sample of autistic and neurotypical individuals (*n* = 776) and extracted 726 variables from each participant’s data. We computed effect sizes and split-half replication rate for group differences between autistic and neurotypical individuals for each EEG variable while accounting for age, sex and IQ. Bootstrapping analysis with different sample sizes was done to establish how effect size and replicability varied with sample size.

**Results:**

Despite the broad and exploratory approach, very few EEG measures varied with autism diagnosis, and when larger effects were found, the majority were not replicable under split-half testing. In the bootstrap analysis, smaller sample sizes were associated with larger effect sizes but lower replication rates.

**Limitations:**

Although we extracted a comprehensive set of EEG signal components from the data, there is the possibility that measures more sensitive to group differences may exist outside the set that we tested. The combination of data from different laboratories may have obscured group differences. However, our harmonisation process was sufficient to reveal several expected maturational changes in the EEG (e.g. delta power reduction with age), providing reassurance regarding both the integrity of the data and the validity of our data-handling and analysis approaches.

**Conclusions:**

Taken together, these data do not produce compelling evidence for a clear neurobiological signature that can be identified in autism. Instead, our results are consistent with heterogeneity in autism, and caution against studies that use autism diagnosis alone as a method to categorise complex and varied neurobiological profiles.

**Supplementary Information:**

The online version contains supplementary material available at 10.1186/s13229-025-00647-3.

## Background

Although the neurobiology of autism is not fully understood, preclinical work implicates differences in neuronal-cortical organisation that cause mechanistic alterations at different levels of the nervous system including synaptic transmission and neural connectivity [2–4]. Building on these findings, a growing literature has sought to identify potential neurobiological differences between autistic and neurotypical individuals.

EEG, specifically resting state EEG (rsEEG), is a particularly useful method in this work given that oscillatory activity computed from rsEEG provides detailed insight into neural dynamics that are, in turn, modulated by many of the markers implicated by preclinical work. For example, high frequency EEG oscillations in the gamma range are shaped by GABA_A_ receptor mediated inhibitory neurotransmission [5], and multiscale entropy (MSE) computed from rsEEG data provides insight into network functional connectivity [6]. These variables can be obtained from very short (< 2 min) paradigms with limited cognitive demands, enabling data acquisition from a wide range of participants including infants and nonverbal individuals [7]. rsEEG is also the most accessible neuroimaging method from the perspective of the participant and can be administered outside of the clinic or laboratory [8] increasing likelihood of clinical translation, e.g. in possible autism screening or biomarker detection. There is currently no recognised EEG biomarker for ASD, yet identifying such a biomarker from rsEEG would revolutionise the diagnosis of autism and provide much sought after insight into the neurobiology of the condition. For this reason, the focus of the work presented here is on variables derived from rsEEG.

rsEEG variables that have been reported most frequently in studies comparing autistic and neurotypical individuals include spectral power across five canonical frequency bands (delta, theta, alpha, beta and gamma) and intersite phase clustering (ISPC), reflecting network connectivity. A recent systematic review of these variables found that significant differences between autistic and neurotypical individuals were reported in all (of 21) studies included in the review, but due to heterogeneity within the results generalisations could not be drawn [9]. Similarly, a meta-analysis of spectral power differences in autism found no significant group differences in the majority of frequency bands, although there was a trend towards reduced alpha power and increased gamma power in autism [10].

Beyond quantification of spectral power and measurement of ISPC, other rsEEG variables reported in autism / neurotypical group differences studies include: MSE; phase-amplitude coupling (PAC); the slope of the aperiodic power spectrum (1/f trend slope); and peak frequency of alpha oscillations. Although the volume of studies reporting these variables is not yet sufficient to support meta-analyses, early evaluation indicates that while some group differences have been reported, they are not always replicated and can be contradictory [11–22]. Thus, the current state of the science is that while rsEEG differences between autistic and neurotypical individuals likely exist, the specific manifestation of these differences is unclear.

There are a number of limitations of work in this area which may hamper the identification of robust group differences. Firstly, studies typically have small samples. For example, the mean sample size of the 41 studies included in the meta-analysis by [10] was 30 autistic and 35 NT participants. The largest sample included 142 autistic and 138 neurotypical participants (data included in the present analysis as the femaleASD sample [23], but 85% of the studies included fewer than 50 autistic individuals. Given the complex and heterogeneous nature of autism, it is likely that samples of this size yield both type 1 and type 2 statistical errors. For example, a recent publication from the EU Aims LEAP consortium with 411 participants found that there were no differences between autistic and NT groups that survived validation testing [24, 25]. Secondly, EEG variables change non-linearly with age and may develop differently in autism. Therefore, failure to replicate findings may be due, in part, to the differing age ranges of the samples tested. Thirdly, most investigations restrict their analyses to a small number of variables, to a subset of electrode locations and, in the case of spectral analyses, to particular frequency bands, which again may contribute to inconsistencies across studies.

Here, we sought to address these limitations by combining data from five previously collected datasets to create a large sample of 776 participants (421 autistic and 355 neurotypical). Data were obtained across a wide age range - from infancy to young adulthood - but were analysed in three distinct age brackets to allow us to detect differences in the neural functioning of autistic individuals at different ages. We took an exploratory and comprehensive approach by computing many of the variables that have previously been suggested as differing between autistic and NT people, including absolute, relative, and log-transformed power, hemispheric power asymmetry, peak alpha frequency, MSE, PAC, ISPC, and 1/f slope. For each variable, we included the full range of frequency bands (delta to gamma) and full electrode coverage across the scalp, computing a total of 726 variables from each participant. In recognition of the risk of type 1 errors, we report effect sizes regardless of p-value, and carry out split-test validation to assess the reliability of these effects. To our knowledge, this is the largest sample in which EEG data between autistic and neurotypical samples has been reported, both in terms of sample size and the number of variables analysed, and the only study to report reliability testing that goes beyond leave-one-out methods. Furthermore, because of the size of the dataset, we were able to carry out exploratory analyses investigating potential interaction effects with age and sex.

Our aim was to provide a comprehensive account of the rsEEG variables that do and do not differ between autistic and neurotypical individuals in order to identify which variables are worthy of future interrogation, particularly for studies that are aimed at understanding the neurobiology of autism and / or pursuing the search for potential EEG biomarkers of autism. However, as the results reported below demonstrate, even when addressing the limitations outlined above, we found little evidence for reliable differences in rsEEG data obtained from the autistic and neurotypical samples.

## Methods

This project was pre-registered [26]. No changes were made to data selection, cleaning, and signal processing steps, and the age group divisions and core statistical model were not changed. However, in response to reviewer comments, many changes to the analysis were made. For ease of exposition, differences are not highlighted throughout the methods and no further mention is made to our pre-registration, but the interested reader can see the evolution of our data analysis methodology from the pre-registration [26], to our pre-print [27], to the present writing. In all cases, new analyses were undertaken to increase statistical rigour, address possible confounds, and increase clarity.

Due to the size of the dataset analysed here, analysis depended on efficient use of high power computing (HPC) resources provided by the University of Sheffield. Please see the supplement for tutorial details regarding structuring analysis code and formatting data for compatibility with HPC analysis.

### Data

No new data were collected for this project. We combined eyes-open rsEEG data from five separately collected datasets. Table 1 displays demographic information and reflects the final dataset after cleaning, and Supplemental Fig. 2 displays the age distribution of participants from different datasets. All raw data were obtained through the National Institute of Mental Health (NIMH) data archive (NDA), and can be accessed there by interested researchers [1]. All participants diagnosed with autism by the original data collection teams were assessed using the Autism Diagnostic Observational Schedule (ADOS), and we split these participants into two groups using the terminology provided by the ADOS: an Autism Spectrum Disorder (ASD) group, and an Autism Disorder (AD) group. For participants too young to be assessed with the ADOS, diagnosis was confirmed with the ADOS retrospectively at ~ 36 months of age [28]. The original data collection teams also recruited neurotypical participants who were assessed as not having autism either using the ADOS or through the judgement of a qualified clinician from the original data collection team. These participants formed a control (CON) group for the present analysis. The motivation for splitting the autism group into an ASD and an AD group was in recognition of the heterogeneity of the condition; ADOS score was the only information available across all participants to support any kind of sub-typing. This severity subtyping did not have a large impact on the interpretation of differences between AD and CON groups (see Supplemental Fig. 4). This sorting was based on standard cut offs as specified in the ADOS manual [29] More details about the participant inclusion/exclusion criteria implemented by the original data collection teams, the data preprocessing, harmonisation, and cleaning can be found in the supplement.

**Table 1:** Demographic and data quality statistics for all participants

group	data set	IQ	IQ metric	age in months	*n* female	*n* total	orig channels	final channels	orig epochs	final epochs
AD	biomarkCon	98 (17; 60–150)	DAS GCA	106 (20; 73–140)	39	168	124 (0)	109 (15)	91 (0; 91–91)	78 (11; 52–91)
ASD	biomarkCon	108 (13; 84–137)	DAS GCA	107 (21; 73–139)	7	23	124 (0)	117 (8)	91 (0; 91–91)	83 (8; 61–91)
CON	biomarkCon	116 (12; 90–155)	DAS GCA	104 (20; 72–142)	35	106	124 (0)	117 (8)	91 (0; 91–91)	85 (8; 52–91)
AD	biomarkDev	71 (24; 36–131)	MSEL	39 (38; 3-133)	10	68	125 (0)	89 (17)	52 (16; 20–100)	40 (13; 12–74)
ASD	biomarkDev	106 (19; 80–137)	MSEL	11 (8; 3–24)	4	10	125 (0)	90 (12)	50 (18; 38–100)	36 (4; 30–42)
CON	biomarkDev	104 (17; 49–135)	MSEL	9 (4; 3–29)	22	50	125 (0)	92 (13)	53 (14; 36–100)	41 (12; 10–76)
AD	femaleASD	99 (20; 62–167)	WTAR	146 (32; 97–214)	41	112	125 (0)	125 (1)	65 (27; 10–123)	65 (27; 10–123)
ASD	femaleASD	103 (21; 68–149)	WTAR	155 (38; 96–215)	25	40	125 (0)	125 (1)	67 (29; 10–121)	67 (29; 10–121)
CON	femaleASD	113 (15; 79–149)	WTAR	156 (35; 96–216)	92	178	125 (0)	125 (1)	80 (23; 20–127)	80 (23; 20–127)
AD	socBrain	99 (10; 87–119)	DAS GCA	233 (9; 216–243)	0	7	62 (0)	62 (0)	154 (4; 150–160)	124 (34; 66–157)
ASD	socBrain	97 (12; 87–110)	DAS GCA	233 (13; 222–248)	0	3	62 (0)	51 (9)	152 (3; 150–155)	101 (44; 52–136)
CON	socBrain	97 (0; 97–97)	DAS GCA	232 (6; 228–236)	1	2	62 (0)	62 (0)	154 (6; 150–159)	132 (23; 115–148)
AD	bpSZ		WTAR		0	0				
ASD	bpSZ		WTAR		0	0				
CON	bpSZ	104 (9; 94–120)	WTAR	216 (21; 180–240)	4	9	62 (0)	59 (3)	100 (0; 100–100)	79 (10; 63–93)

DAS GCA = Differential Ability Scales General Conceptual Ability

MSEL = Mullen Scales of Early Learning

WTAR = Weschler Test of Adult Reading

biomarkCon = The Autism Biomarkers Consortium for Clinical Trials

biomarkDev = Biomarkers of Developmental Trajectories and Treatment in ASD

bpSZ = Bipolar & Schizophrenia Consortium for Parsing Intermediate Phenotypes

femaleASD = Multimodal Developmental Neurogenetics of Females with ASD

socBrain = The Social Brain in Schizophrenia and Autism Spectrum Disorders

Numeric values indicate mean and (standard deviation; min-max)

### Extraction of key EEG variables

Power spectra, 1/f trend slope, peak alpha frequency (PAF), phase-amplitude coupling (PAC), multiscale entropy (MSE), and intersite phase clustering (ISPC) were all calculated using standard methods. For details, please see supplemental methods. Briefly, power spectra were extracted by averaging narrow band filtered power time series across both time and epoch. Narrow band filtering was done using gaussian convolution in the frequency domain. 1/f exponent and offset values were extracted using the FOOOF package in python [30]. PAF was calculated by taking the mean of the best fitting gaussian in a candidate range of 6 to 14 Hz [13]. PAC was calculated following methods presented by Tort et al. [31] with frequency bands suggested by Peck et al. [14]. MSE was extracted by varying the graining of the data and then calculating the entropy at various graining levels [32, 33], and ISPC was calculated on Laplacian transformed data using methods described by [34].

### Channel groupings for comparisons

All calculations generated values for every data channel (except ISPC, which generated values for pairs of channels), and for 100 frequencies. However, signals at adjacent channels and adjacent frequencies are correlated. In addition, utilising all calculated values as dependent variables would be intractable (e.g. 32 channels X 100 frequencies = 3200 raw power variables alone). Thus, variables were averaged into 13 regional groups (right frontal, left frontal, right centroparietal, left centroparietal, right occipito parietal, left occipito parietal, frontal, occipital, central, left lateral, right lateral, right hemisphere, and left hemisphere) and five asymmetry sensitive comparisons (difference scores between groups of electrodes: interhemispheric, rostrocaudal difference within the left and right hemispheres, and mediolateral difference within the left and right hemispheres). This yielded 18 total groupings of channels. Data were further averaged into 6 canonical frequency bands: δ (2–4 Hz), θ (4–8 Hz), α (8–14 Hz), 𝛽 (14–30 Hz), 𝛾low (30–50 Hz), and 𝛾high (50–80 Hz). See supplemental material for the specific variable averaging schemes.

In total there were 726 (324 power + 36 1/f slope + 36 peak alpha + 216 PAC + 72 MSE + 42 ISPC) dependent variables.

### Statistical evaluation of group differences for every dependent measure

Data were divided into tertiles using age such that there were an equal number of AD and ASD participants in each group. This resulted in age groups consisting of 3 to 96 months old, 98 to 126 months old, and 128 to 248 months old participants, see Table 2 for descriptive statistics for each age group. This was done because it was possible that there would be complex interactions between age and autism diagnosis that would require bespoke modelling of each EEG variable. Splitting data into age groups made the analysis more sensitive to effects that might be specific to particular age ranges. Indeed, our results revealed group differences in different EEG variables in different age groups. To account for non-linearity in age effects, all EEG measures were regressed onto age as a single predictor and the combination of age and age squared:

**Table 2:** Age (in months) and number of participants in the three age groups

group	age min	age max	*n* AD	*n* ASD	*n* CON
Youngest	3	96	123 (26)	19 (7)	93 (36)
Middle	98	126	115 (31)	21 (9)	84 (42)
Oldest	128	248	108 (29)	35 (19)	161 (73)

Parenthetical values indicate the number of female participants in each group

$$\eqalign{& Age\,alone\,model: \cr & EEG\,dependent\,measure\, \sim \,Age \cr} $$

$$\eqalign{& Age\,quadratic\,model: \cr & EEG\,dependent\,measure\, \sim Age\, + \,Ag{e^2} \cr} $$

A likelihood ratio test (lrtest in R) was used to compare these two nested models. The null hypothesis for this test was that the coefficient of the $\:{Age}^{2}$ term was zero. In other words, for EEG variables where the likelihood ratio test was significant, it indicated that the true value of the coefficient of the $\:{Age}^{2}$ term was not zero, and so $\:{Age}^{2}$ was used as an additional predictor in models M1-M4 for these EEG variables. Doing this helped to account for quadratic changes in some EEG variables as a function of age.

For our main analysis, all EEG measures were regressed onto the $\:Age,\:Sex,\:IQ,$ and $\:Diagnosis$ variables using the following models fit with the lm function in R:

$$\eqalign{& M1:\>EEG\,dependent\,measure \cr & \sim Age + Sex + IQ + Diagnosis \cr} $$

$$\eqalign{& M2:\>EEG\>dependent\>measure\> \sim \cr & Age + Sex + IQ + Diagnosis + Diagnosis*Sex \cr} $$

$$\eqalign{& M3:\>EEG\>dependent\>measure\> \sim \cr & Age + Sex + IQ + Diagnosis\> + \>Diagnosis*Age \cr} $$

$$\eqalign{& M4:\>EEG\>dependent\>measure\> \sim \cr & Age*Sex*Diagnosis + IQ \cr} $$

These models were fit independently for each EEG measure and age group resulting in 8712 statistical tests. $$\:{{\eta\:}_{partial}^{2}}^{}$$was extracted to measure effect size for the following independent variables in model M1: $$\:Age,\:Sex,\:IQ,$$ and $$\:Diagnosis$$. From models M2, M3, and M4, $$\:{{\eta\:}_{partial}^{2}}^{}$$associated with the following interaction terms was extracted: $$\:Diagnosis*Sex$$, $$\:Diagnosis*Age$$, and $$\:Age*Sex*Diagnosis$$, respectively. In all cases, type III sums of squares was used to calculate $$\:{\eta\:}_{partial}^{2}$$. Using type III sums of squares means that all effect size values reflect the effect of a predictor assuming that all other predictors were added into the model before it.

### Assessing effect size stability

Due to the exploratory nature of the analytical approach, we expected a substantial number of false positive coefficients in our results. To test the stability of these coefficients, we used bootstrapped split-half cross-validation on all models where individual coefficients were associated with $\:{\eta\:}_{partial}^{2}$ above 0.035. This procedure followed multiple steps, where we: (1) split the data into two halves, making sure that both halves had an equal proportion of participants in each diagnostic group and distributions of age and sex, (2) refit models with large effect sizes to data splits and recalculated the effect size values, (3) repeated this procedure for 150 random splits of the data and calculated the proportion of splits for which both halves exhibited $\:{\eta\:}_{partial}^{2}$ above 0.035 for each variable. We termed this proportion the replication rate. We identified variables with a replication rate of at least 0.64. The 0.64 level corresponds to the standard convention of 0.80 power. That is, if statistical tests are deemed ‘good’ when they detect an effect with probability 0.8, then the implication is that $\:{0.8}^{2}=0.64$ is an acceptable level of consistency for the replication rate when a test is repeated. The $\:{\eta\:}_{partial}^{2}$ cut off of 0.035 was chosen because this is between the standard guidance for small and moderate effect sizes [35] and effects smaller than this would be unlikely to be of interest. In addition, this was a level at which the p-value was robustly below 0.05 (Fig. 2d).

## Results

### Qualitative assessment of the data

**Fig. 1:**
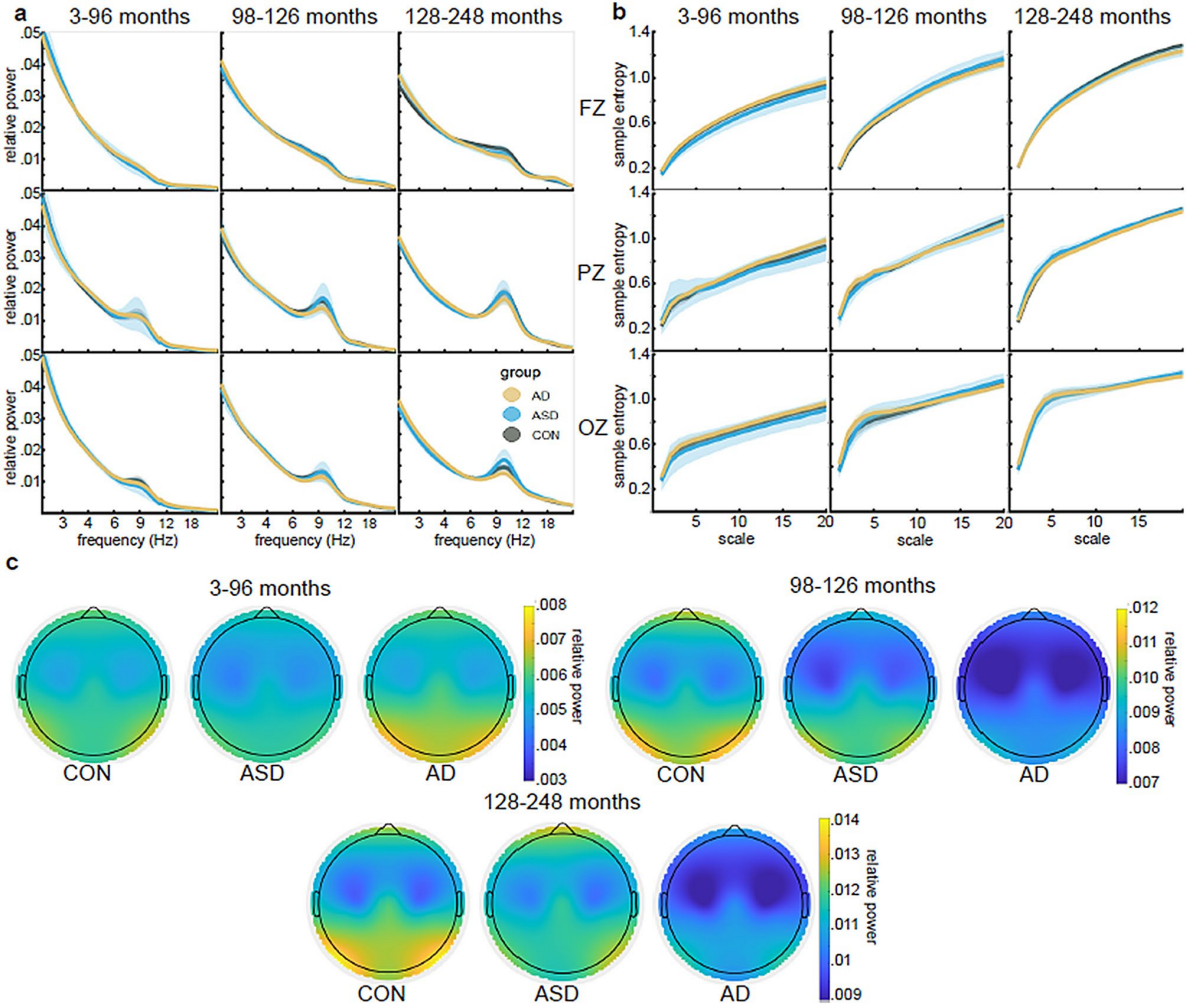
Example group means of selected dependent variables. **a**. Group mean power spectra are displayed in units of relative power. The rows correspond to different electrodes. The columns correspond to age groups. Electrodes and age groups are labelled. Diagnosis group is indicated in colour. Shaded regions indicate the 95% confidence interval. **b**. Group mean multiscale entropy (MSE) values are displayed. Rows and columns are organised similarly to panel a. c. Topographical maps of spectral power in the alpha band (7.5–14 Hz) are displayed for the three age and diagnosis groups. Each plot is scaled independently to display maximum contrast. However, as can be seen in panel a, there was not a great deal of variability between diagnostic groups in the alpha band, and differences were largely contained to increasing alpha power with age

Figure 1 displays group averaged power spectra and multi-scale entropy within each age group at electrodes Fz, Pz, and Oz and group average topographical maps of alpha power within each age group and for each diagnosis. Visual inspection of these plots indicates that indices appear as expected in terms of topographical distribution of power, shape of power spectra and relationship between entropy and scale, providing reassurance regarding the integrity of the data and the appropriateness of the analysis approach on data that were obtained from several sources.

### Quantitative search for group differences

Inclusion of a non-linear effect of age in predicting the EEG provided a significant improvement in model fit for 24.5% of EEG variables. For these variables, an $\:{Age}^{2}$ term was added to subsequent models.

Extreme outliers were removed prior to modelling for each EEG variable (see supplemental methods for detailed description of outlier detection procedure). For 42.5% of all variables there were zero outliers. The maximum number of outliers was 40 observations for any single EEG variable. The mean number of outliers removed from each analysis from neurotypical participants was 3.9 and the corresponding value for autistic outliers was 3.6.

**Fig. 2:**
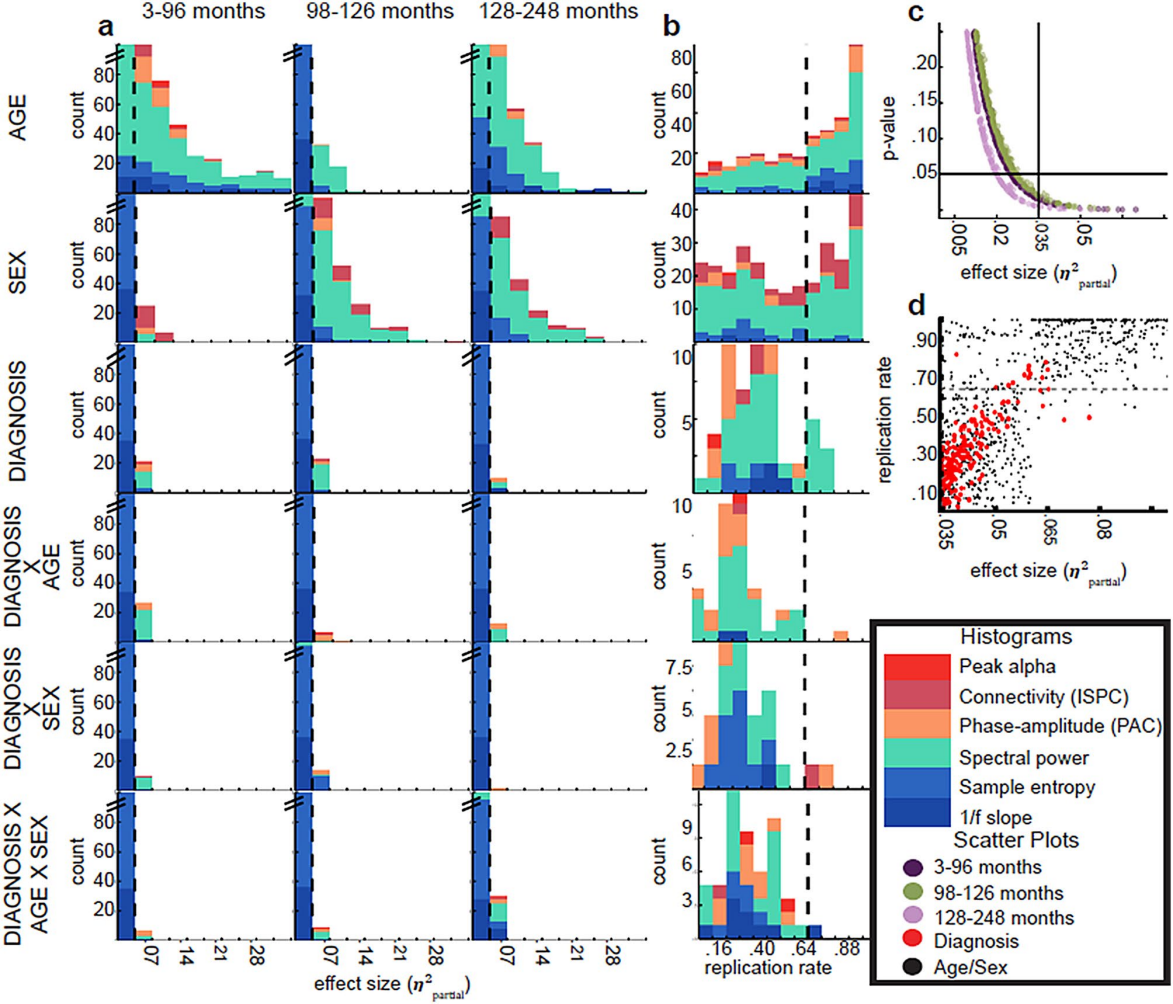
Diagnosis predicted few EEG variables with both high replication rate and effect size. **a**. Histograms display 𝞰²partial values associated with prediction of EEG dependent variables by various independent variables (vertical axis of panels) in different age groups (horizontal axis of panels). Colours indicate different categories of EEG variables. All y axes are truncated at 100. Vertical dashed lines indicate the threshold of 𝞰²partial = 0.035. **b**. Histograms display the replication rate of the ability of various independent variables (vertical axis of panels) to predict EEG dependent variables with 𝞰²partial > 0.035. Replication rate was obtained by bootstrapping random half splits of the data and asking what proportion of splits yielded 𝞰²partial > 0.035 in both halves. Colours indicate different categories of EEG variables. Data are collapsed across age groups for visualisation. Vertical dashed lines indicate the threshold of replication rate = 0.64. **c**. The scatter plot displays the relationship between 𝞰²partial and p-value for diagnosis coefficients. Lines indicate the threshold of *p* =.05 and 𝞰²partial = 0.035. Colours indicate age groups. d. The scatter plot displays the relationship between 𝞰²partial and replication rate. Black dots indicate values for age and sex coefficients. Red dots indicate values for diagnosis, diagnosis*age, diagnosis*sex, and diagnosis*age*sex coefficients. The horizontal dashed line indicates the threshold of replication rate = 0.64. Diagnosis was able to predict 13 variables with replication rate > 0.64 and 𝞰²partial > 0.035. The trend exhibited in the plot suggests that lowering the 𝞰²partial threshold would have resulted in very low replication rates

Figure 2a displays histograms of $\:{\eta\:}_{partial}^{2}$values for each independent variable across age groups for all of the above models. Table 3 displays the proportion of EEG variables predicted with $\:{\eta\:}_{partial}^{2}$>0.035 split by EEG variable type (e.g. spectral power, ISPC, MSE, PAF or PAC) and for each independent variable type separately (age, sex, diagnosis, IQ and their interactions). In total, just over 2% (*N* = 54) of all diagnosis coefficients (*N* = 2,184) had $\:{\eta\:}_{partial}^{2}$>0.035. The corresponding value for interactions between diagnosis and age, sex, or age X sex was 1.8%. By contrast, 28% of the age coefficients and 20% of sex coefficients had $\:{\eta\:}_{partial}^{2}$>0.035. The adjusted generalised standard error inflation factor associated with diagnosis was never higher than 1.2 in any model, suggesting that multicollinearity did not have an impact on the results presented here [36] (see also supplemental extended multicollinearity analysis and supplementary Fig. 2c and d).

**Table 3:** Performance of different predictors in each age group for different dependent variables (DV)

Age Group	DV	Age	Sex	IQ	Diagnosis	Diag*Age	Diag*Sex	Diag*Age*Sex
3–96 months	PAC	0.18	0.02	0.01	0.02	0.02	0	0.02
3–96 months	PAF	0.32	0	0	0.03	0	0	0
3–96 months	ISPC	0.38	0.5	0	0.02	0	0.02	0
3–96 months	power	0.64	0.02	0.06	0.03	0.06	0.02	0.01
3–96 months	MSE	0.81	0	0	0.03	0	0	0
3–96 months	slope	0.69	0	0	0.03	0.06	0.03	0.03
98–126 months	PAC	0	0.04	0.01	0.01	0.02	0.01	0.01
98–126 months	PAF	0	0.03	0	0	0.03	0	0.03
98–126 months	ISPC	0	0.83	0.02	0.05	0.02	0	0
98–126 months	power	0.14	0.43	0.02	0.05	0	0	0.02
98–126 months	MSE	0.07	0.17	0	0.03	0	0.14	0.01
98–126 months	slope	0	0.11	0	0	0	0	0
128–248 months	PAC	0.11	0	0	0.01	0.02	0.01	0.01
128–248 months	PAF	0.05	0	0	0	0	0	0.03
128–248 months	ISPC	0.17	0.76	0	0	0	0	0.02
128–248 months	power	0.44	0.37	0	0.01	0.03	0	0.04
128–248 months	MSE	0.53	0.31	0	0	0	0	0.07
128–248 months	slope	0.53	0.03	0	0.08	0	0	0.22
MEAN	--	0.28	0.2	0.01	0.02	0.01	0.01	0.03
SD		0.27	0.27	0.01	0.02	0.02	0.03	0.05

Proportion of independent variables predicted with partial 𝜂²0.035

PAC = phase amplitude coupling; PAF = peak alpha frequency; ISPC = intersite phase clustering; power = spectral power; MSE = multiscale entropy; slope = 1/f trend slope

### Group differences that were replicable

Figure 2b displays histograms of the replication rate for each independent variable collapsed across age groups and only for effects with $\:{\eta\:}_{partial}^{2}$>0.035. For both age and sex, these histograms were skewed towards large replication rates. By contrast, for diagnosis, the distribution of replication rate values was concentrated towards low values, indicating poor replicability of effects. In general, the replication rate was higher for larger $\:{\eta\:}_{partial}^{2}$ values (Fig. 2e; supplemental Fig. 2a) across all independent variables.

**Fig. 3:**
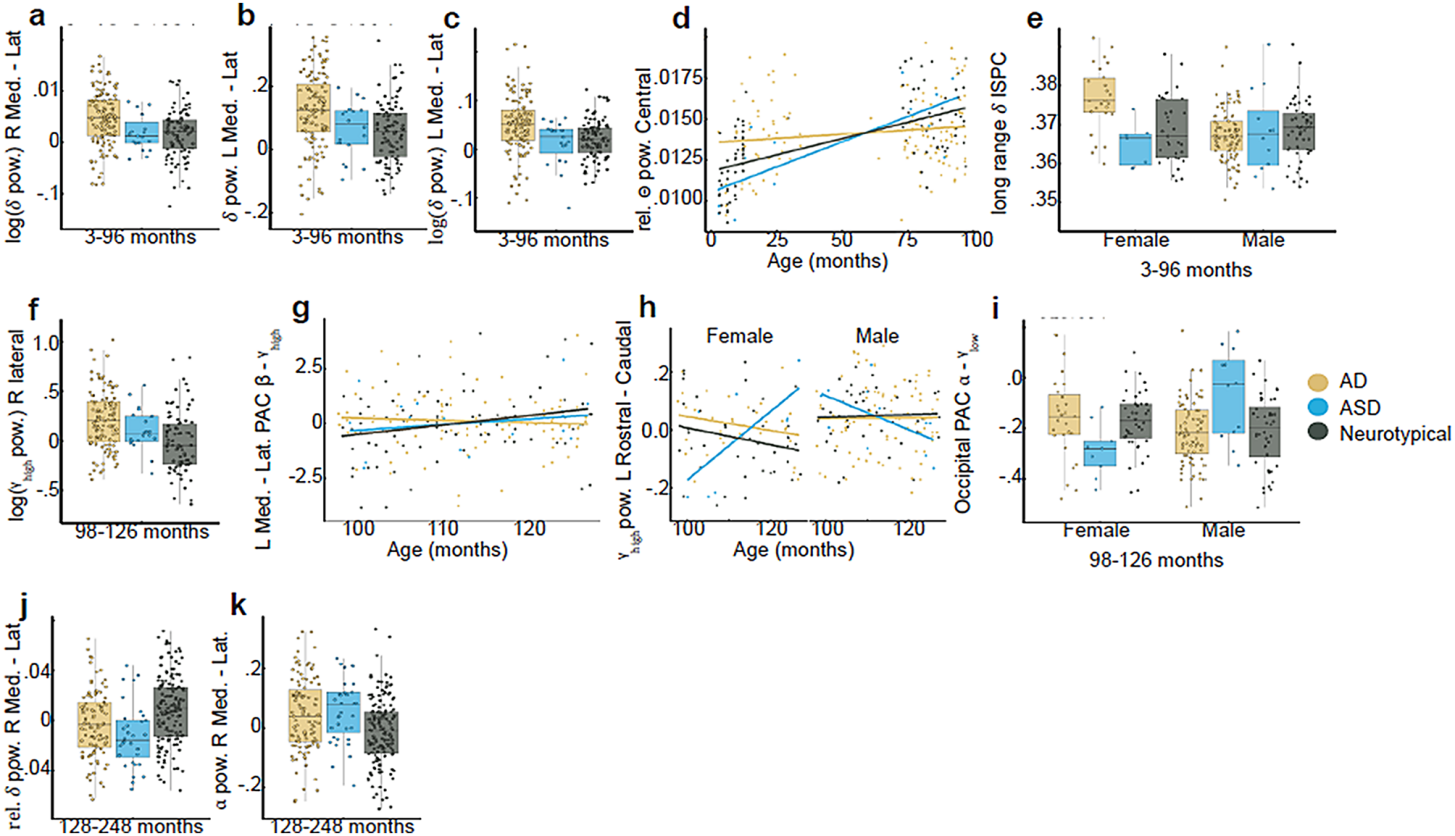
11 EEG variables were well predicted by diagnosis or its interaction with age, sex, or age and sex. Across panels, dots represent individual participants. For box plots, vertical lines extend to 1.5 times the interquartile range above and below the 75th and 25th percentile of the data. The box indicates the 25th to 75th percentile with the median marked by a horizontal line. For scatter plots, lines indicate linear best fits to the data. Panels **a-e** display effects from the youngest age group. Panels **f-i** display effects from the middle age group, and panels **j-k** display effects from the oldest age group

Considering diagnosis specifically, 11 of the 174 coefficients with $\:{\eta\:}_{partial}^{2}$> 0.035 yielded a replication rate > 0.64. These variables are listed in Table 4 and plotted in Fig. 3. Five of these variables were in the youngest age group, four were in the middle age group, and two were in the oldest age group. Interestingly, across all age groups, 7 of 11 variables were asymmetry measures. In addition, 7 of 11 were variables measuring aspects of lower frequency signals, i.e. in the delta, theta, and alpha bands.

**Table 4:** Variables that passed both effect size and replication rate thresholds

dependent Variable	Age Group	IV	$\:{\eta\:}_{partial}^{2}$	replication rate
central relative theta power	1	Diag * Age	0.06	0.65
Asymmetry in delta power left hemisphere midline - lateral	1	Diag	0.06	0.71
Asymmetry in log delta power left hemisphere midline - lateral	1	Diag	0.06	0.67
Asymmetry in log delta power right hemisphere midline - lateral	1	Diag	0.07	0.71
long range delta ISPC	1	Diag * Sex	0.06	0.72
log of high gamma power in right lateral	2	Diag	0.06	0.74
asymmetry in high gamma power left hemisphere rostral - caudal	2	Diag * Age * Sex	0.07	0.66
Alpha - gamma PAC occipital	2	Diag * Sex	0.06	0.85
Asymmetry in beta - high gamma PAC left hemisphere midline - lateral	2	Diag * Age	0.04	0.84
Asymmetry in relative delta power right hemisphere midline - lateral	3	Diag	0.05	0.65
Asymmetry in alpha power right hemisphere midline - lateral	3	Diag	0.06	0.79

### The effect of sample size on and replicability

**Fig. 4:**
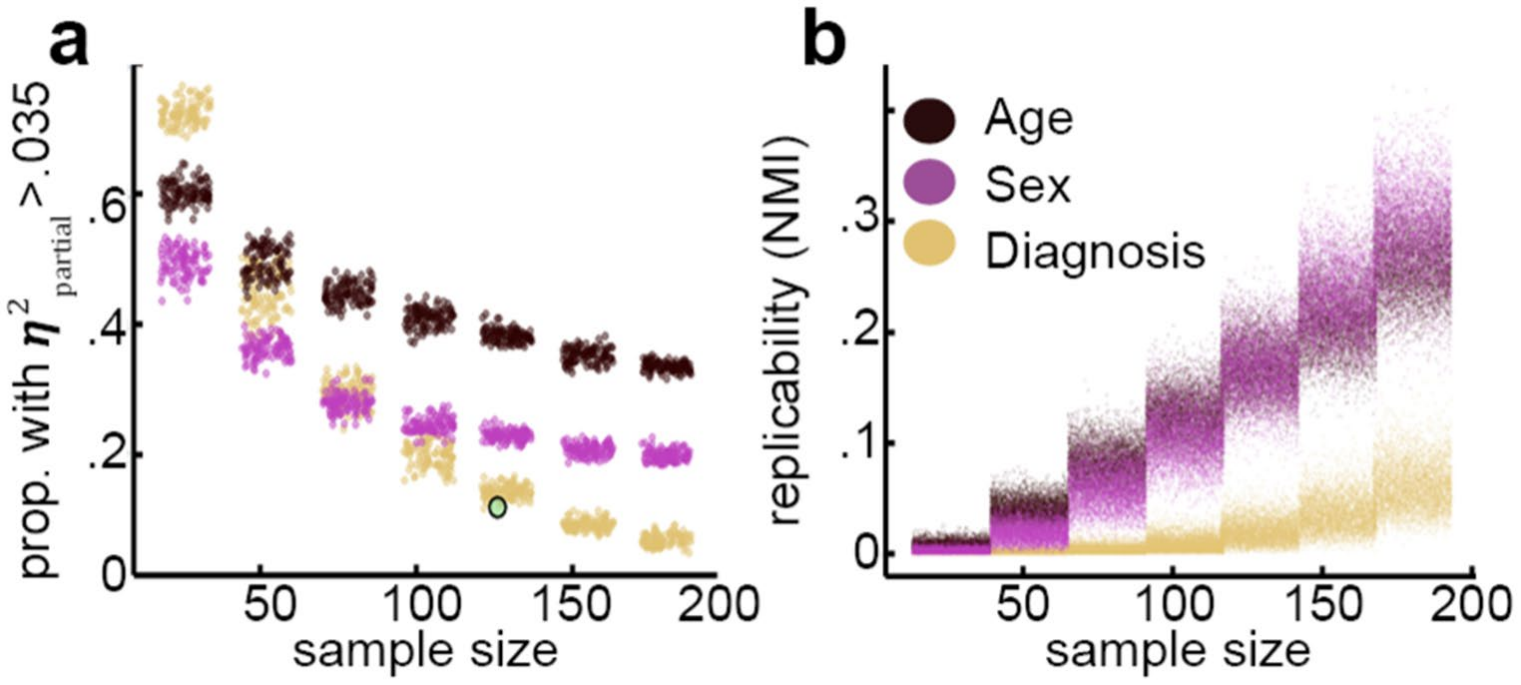
Small sample sizes produced more high effect sizes but low replicability. 100 random samples for each of 7 different sample sizes were taken from each age group without replacement. Results are shown here collapsed across age groups. Colours indicate the independent variable. **a**. The scatter plot displays the relationship between the proportion of EEG variables predicted with 𝞰²partial > 0.035 (y axis) and sample size (x axis). Each dot represents the results obtained from one random sample. Notice that the proportion of EEG variables predicted with a meaningful effect size drops much more quickly for diagnosis as a predictor than for age or sex. The green dot denotes the results of an analysis in which a demographically matched sample was constructed. **b**. The scatter plot displays the relationship between the replicability (measured as normalised mutual information; y axis) and sample size (x axis). Each dot represents the NMI in the pattern of EEG variables predicted with 𝞰²partial > 0.035 between two random samples of the data. Notice that as sample size rises, the replicability of results obtained for age and sex predictors rises faster than for diagnosis

In order to investigate whether sample size per se has a bearing on the likelihood of finding differences in rsEEG between autistic and neurotypical samples, we repeated our entire analysis 100 times for each of 7 different sample sizes (10%, 20%, 30%, 40%, 50%, 60%, and 70% of the total sample size available for each age group). On each repetition, data were randomly sampled without replacement such that the proportion of participants in each diagnosis group and sex was the same as in the total dataset for each age group. Across bootstrap samples, two key statistics were extracted. First, the proportion of $\:{\eta\:}_{partial}^{2}$ values greater than 0.035 was calculated for every bootstrap sample. Second, normalised mutual information (NMI) for the pattern of EEG variables associated with $\:{\eta\:}_{partial}^{2}$ greater than 0.035 was used to assess replicability [37, 38]. NMI was calculated for every possible pair of bootstrap samples using the NMI function in the randnet R package. This approach demonstrated that when sample size was low, diagnosis predicted the EEG with $\:{\eta\:}_{partial}^{2}$ greater than 0.035 more often than did age or sex (Fig. 4a). As sample size increased, the percentage of EEG variables that were well-predicted by diagnosis decreased and approached 0% as sample size approached 200. By contrast, age and sex approached asymptotes as sample size increased, predicting approximately 40% and 20% of EEG variables, respectively.

NMI values indicated that not only were fewer EEG variables predicted by diagnosis than by age and sex, but the set of EEG variables predicted by diagnosis was also less replicable (Fig. 4b). Specifically, as sample size increased, NMI increased more slowly for diagnosis than for either age or sex.

Finally, the possible effects of multicollinearity were examined one more time. Since multicollinearity is systematic dependencies between independent variables, the best way to eliminate its effects is to remove any such dependencies by rigorously matching samples. Thus, for each AD and ASD participant, a CON participant was individually selected with the same sex, an age within 5 months, and an IQ within 10 points. Any AD or ASD participant for whom no match was available was discarded. Any CON participant not used to match with an AD or ASD participant was discarded. This procedure made multicollinearity mathematically impossible, but it reduced the available sample size. The green dot in Fig. 4a represents the sample size and observed proportion of EEG variables predicted with $\:{\eta\:}_{partial}^{2}$ greater than 0.035 in this matched sample. If it were the case that multicollinearity were artificially holding down $\:{\eta\:}_{partial}^{2}$ estimates associated with diagnosis in the non-matched data, then creating a matched sample should have revealed a sudden rise in $\:{\eta\:}_{partial}^{2}$ associated with diagnosis. However, no rise was observed, suggesting that multicollinearity did not play a role in any of the analyses presented here.

## Discussion

The search for reliable differences in rsEEG between autistic and neurotypical individuals is a growing area of research but has not yet yielded clear outcomes. Limitations of existing work include small samples and limited selection of variables reported. Here, we pooled data from five existing studies to assemble a large dataset of 776 individuals, 421 of whom were diagnosed with autism. We took an exploratory approach, applied a standardised pipeline to harmonise and clean the data and used established methods to extract multiple variables that have previously been hypothesised to differ between autistic and neurotypical samples.

At first glance our analyses provide support for differences in rsEEG obtained from autistic and neurotypical samples as we found 174 measures of EEG dynamics which could be predicted by diagnosis or its interaction with sex or age with an effect size of at least 0.035. As shown in Table 3, these models included variables involving all the different types of dependent variables, in line with published data. However, when we tested the validity of these differences using split-half analysis, the vast majority of these effects were less than 64% likely to replicate in both halves of the data. To our knowledge, this is the first study to test the replicability of identified group differences in EEG variables computed from autistic and neurotypical participants within the same sample. The low replication rate obtained via split-half testing is aligned with the literature as a whole which shows generally low levels of replication between different studies (for example, see 9). We carried out analyses to confirm that the data generated expected outcomes in order to reassure ourselves of the integrity of our secondary-data analysis approach. These analyses confirmed that this particular dataset replicated previous findings with respect to the range of values obtained for key variables and the topographical distribution of alpha power across the scalp (see Fig. 2). We also replicated established findings including age-related increase in MSE [39] and age-related decrease in theta and delta power [40–42] (see Supplemental Fig. 3a-c). Replicating these established findings with this dataset and finding large effect sizes that strongly replicate in models that use Age and Sex as predictors (Fig. 1b) provided reassurance regarding both the integrity of the data and the validity of our data-handling and analysis approaches.

When all data were analysed collectively and not within age boundaries, Diagnosis predicted none of the EEG variables with an effect size greater than 0.035. By contrast, age and sex continued to predict 40% and 16% of EEG variables respectively (see supplemental extended sample size analysis). These results confirm the bootstrap results (see Fig. 4a) which showed that as sample size increased, the number of Diagnosis effect sizes greater than 0.035 decreased and approached 0 as sample size approached 200. Indeed, meta-analysis has revealed that small sample sizes are sometimes associated with higher heterogeneity in effect size [43]. The number of Age and Sex effect sizes greater than 0.035 were also very similar to those found by bootstrapping (Fig. 4a), suggesting true non-zero asymptotes for these variables.

Taken together, these findings have important implications for studies aimed at searching for autism biomarkers. While identifying easily measured, univariate biomarker(s) for autism would undoubtedly bring about a paradigm shift in the way that autism is understood and diagnosed, our work suggests that such biomarkers may not exist. A common refrain in the literature is that autism is highly heterogeneous [25, 44] and that the diagnostic label of autism may represent a constellation of unique genetic and neurological conditions [45]. Despite this, studies continue to compare groups based solely on the presence or absence of an autism diagnosis.

Our findings of very few replicable large effect sizes, and the bootstrap analyses which showed that larger samples generate *fewer* group differences than smaller samples, speak strongly to heterogeneity in the autism sample and cautions against the design of future studies that investigate group differences in neural markers when groups are classified on the presence or absence of an autism diagnosis alone. Instead, a more profitable line of enquiry is likely to emerge by investigating neural markers of particular features of the autism phenotype in line with the RDOC approach [46], or within more homogeneous subgroups of autistic individuals, e.g. based on stratification to create subgroups of participants who share particular sets of characteristics or genetic markers [47]. Recent work has demonstrated that grouping participants by differences in CNV can more effectively predict fMRI connectivity than grouping the same participants by mental health diagnosis [48]. By extension, clearer neural differences in rsEEG data might be seen when groups are based on a known CNV rather than on a diagnosis, such as autism, where potential genetic variance is unknown. For example, more consistent electrophysiological network alterations were found in a group of participants with a deletion of a specific CNV (22q11.2) than in a group of participants with heterogeneous CNVs [49]. However, within this same study, some findings, such as decreased network connectivity, were common across genotypes suggesting that a “genetics first” approach may not entirely eliminate heterogeneity. Alternatively, where fine-grained behavioural phenotypic data are available, it may be possible to correlate different patterns of rsEEG dynamics with behavioural subtypes, as has been shown for depression [50].

Notwithstanding the above, we found thirteen variables that may be worthy of future investigation given their effect size and replication rate (see Table 4). In line with our position regarding the value of stratifying samples, these findings suggest that group differences may be moderated by age, by sex, by ADOS score and / or by a combination of the above. For example, autistic participants in the youngest age group exhibited consistently higher medial relative to lateral delta power on both the right and left side of the head (Fig. 3a, b,c), but in the oldest age group, this pattern reversed such that neurotypical participants exhibited higher medial relative to lateral delta power at right lateralized scalp locations (Fig. 3j). We also found an interaction between age and diagnosis in their prediction of central theta power in the youngest age group. This indicated that while infants in the AD group exhibited increased theta power over central scalp locations relative to neurotypical infants, by the age of 75 to 100 months of age, this pattern had reversed. Finally, there was an interesting interaction between sex and long-range delta connectivity in the youngest age group (Fig. 3e). Specifically, AD group females exhibited increased long range delta connectivity relative to all other groups. Visual inspection of the other interaction effects reveals that some of them may have been driven by the ASD group, which had few participants, and so these results should be interpreted with caution (Fig. 3h, i). Furthermore, none of these results were predicted and so await replication.

### Limitations

Because of its increased potential for clinical translation due to its more accessible method, we focused on univariate variables derived from rsEEG data. We therefore cannot rule out the possibility that clearer EEG differences between autistic and neurotypical individuals may manifest during task engagement or in multivariate analyses. Neither can we rule out the possibility that there are further variables that could be extracted from the rsEEG signal that may more successfully differentiate the autistic from the neurotypical sample. For example, it may be that analysis focused on variability rather than mean values could reveal group differences that were not discovered in the present analysis. However, our analysis approach was comprehensive and included indices that represent fundamental features of neural dynamics, therefore it would be somewhat surprising for a novel measure to demonstrate meaningful group differences. In our analyses, we accounted for linear and non-linear effects of age on the EEG and checked for interactions between age and diagnosis when predicting EEG dynamics. However, if sufficient sample sizes were available in a narrow age range, then it would be preferable to compare diagnostic groups without the need for age in the model. Lastly, it is possible that combining collected from multiple labs obscured meaningful group differences. However, this is unlikely as the results replicated several age-related prior results.

### Conclusions

In summary, we conclude that there is limited evidence for differences in rsEEG associated only with a diagnosis of autism. Our lack of convincing group differences is unlikely to be due to the sample sizes being too small or by taking a selective approach to the particular variables being analysed. Instead, we speculate that the lack of group differences is likely due to heterogeneity within the samples and the diagnostic label of autism not being refined enough to capture distinct neural profiles. Future work may find that rsEEG data can better predict particular (potentially transdiagnostic) symptom domains rather than the diagnostic category per se, that reliable group differences are more likely to be observed when comparing neural dynamics in groups defined by genotype or homogenous phenotype rather than by diagnostic label, and / or that alteration in the developmental trajectory of specific variables, e.g. as obtained via longitudinal studies, provides clearer findings than a cross-sectional snapshot. Our results strongly encourage adoption of these types of methodological approaches and caution against the value of designing further studies that use samples defined by autism diagnosis alone to investigate potential neurobiological differences associated with the autism phenotype.

## Electronic supplementary material

Below is the link to the electronic supplementary material.

Supplementary Material 1

## Data Availability

All EEG variables extracted from the raw data are available at the individual participant level at in the Github repository, https://github.com/adede1988/SheffieldAutismBiomarkers.git. All raw data are available through application to the NDA [1]. Custom R and Matlab scripts used in the analysis are available in the same Github repository.
