# Supplementary material for: Exploring EEG resting state differences in autism: sparse findings from a large cohort
Source: Mol Autism. 2025 Feb 24;16:13. doi: 10.1186/s13229-025-00647-3 (PMC11853566; doi:10.1186/s13229-025-00647-3)
Supplement: Supplementary file 1 — Supplementary Material 1 [file 13229_2025_647_MOESM1_ESM.pdf]

Supplemental methods and results for:
Exploring EEG Resting State Differences in Autism: Sparse Findings from a Large Cohort

AUTHORS: Adam J.O Dede, Wenyi Xiao, Nemanja Vaci, Michael X Cohen, Elizabeth Milne

Matlab functions referenced throughout this supplement can be found at https://github.com/adede1988/SheffieldAutismBiomarkers.git.

Data Description

No new data were collected for this project. We combined data from 5 separately collected datasets. Table 1 reflects the final dataset after cleaning, and Supplemental Table 1 provides further detail about data collection and inclusion/exclusion criteria of participants (Hyatt et al., 2022; Marin et al., 2020; McPartland et al., 2020; Neuhaus et al., 2021; Ozonoff et al., 2024; Tamminga et al., 2014). All raw data were obtained through the National Institute of Mental Health (NIMH) data archive (NDA), and can be accessed there by interested researchers (Dede, 2023). With the exception of the bpSZ dataset, all autistic participants were evaluated using the ADOS module appropriate to their age and language ability. Neurotypical participants were assessed using either the ADOS or the judgement of qualified clinicians in the data collection teams. For this analysis, only data from control participants in the bpSZ dataset were considered. These participants were deemed not to have any major psychological disorders by the clinicians involved in the bpSZ dataset's collection. For participants whose EEG had been collected multiple times, only their first EEG dataset was considered. Only participants with eyes open resting state data were included. For participants with both eyes open and eyes closed data, only eyes open data were included. All participants were sorted into one of three groups based on ADOS score and defined using the terminology provided by the ADOS: an Autism Spectrum Disorder (ASD) group, a more severe Autism Disorder (AD) group, and a control (CON) group. This sorting was based on standard cut offs as specified in the ADOS use manual (Lord et al., 2012). Across datasets, after applying the above inclusion/exclusion criteria, there were 1040 participants. In the preregistration, we had planned to analyse data from participants across the entire lifespan. However, there were relatively few observations of individuals with autism above the age of 250 months (380 autistic individuals less than or equal to 250 months old; 12 autistic individuals greater than 250 months old). Thus, analysis was limited to participants less than or equal to 250 months old, leaving 808 participants. Also different from the pre-registered plan was the division of the data into age groups. Specifically, for analysis, participants were split into 3 age groups, and the cut offs between these age groups were determined such that one third of the AD and ASD participants were included in each group. This was done because several EEG metrics as a function of age revealed non-linearity that would have required tailoring the modelling approach for each variable. Within age groups all EEG-age relationships were well-described using a linear model.

NDA global unique identifiers were used to confirm that all subjects contributed only a single experimental session to the present analysis.

Our pre-registration did not anticipate missing IQ data, but for 9 participants, IQ data were missing. For these participants, a value equal to their dataset and diagnosis group mean was substituted.

Data Preprocessing

All data were obtained in a raw state with the exception of the female ASD data set, which was provided on the NDA having already been high pass filtered at .1 Hz, low pass filtered at 100 Hz, and notch filtered at 60 Hz. In addition, channels recorded from electrodes with impedances over 200 kOhm had already been deleted and data were split into 2.048 second epochs by the original data collection team.

For all participants, data were segmented into 2 second epochs (female ASD set left as 2.048 s epochs). Each epoch was forward and backward reflected before being high pass filtered at .5 Hz (matlab function: highpass), low pass filtered at 200 Hz (matlab function: lowpass), and notch filtered at 60 or 50 Hz (depending on country of data collection) to eliminate line noise (matlab builtin functions: iirnotch and filtfilt).

The difference between the maximum and minimum voltage in a moving 80 ms window was calculated for each channel and epoch. Epochs where at least one 100 μV deflection was detected were flagged as potentially noisy. A channel was deemed bad if it crossed the 100 μV threshold in 50% of trials. Bad channels were removed. A trial was deemed bad if 25% of the remaining channels exhibited threshold crossings. Bad trials were removed. These parameters were modelled on the preprocessing steps used in the female ASD data set, which was provided precleaned. In deviation from our pre-registration, we added two data rejection criteria to reject entire participants whose data was deemed too noisy. First, participants whose original data as it came to us included fewer than 20 channels were removed from further analysis. This criterion eliminated one participant. Second, participants for whom 50% or more of channels were rejected were removed from further analysis. This criterion eliminated 31 participants. Thus, the final dataset used for all further analyses included 776 participants. For these participants, the mean and standard deviation of the numbers of trials and channels per participant before (original) and after (final) cleaning are displayed in Table 1. The final numbers of participants included in all groups are displayed in Table 2.

Finally, all data were re-referenced to an average reference and interpolated to a standard 32-channel montage. Interpolating all data to a standard montage facilitated comparison between data collected using variable numbers of electrodes.

For more detail about data import and cleaning steps, see functions readEEGdat.m, removeNoiseChansVolt.m, and convertCoordinates.m.

Computation of key EEG variables

Power spectra:

Power spectra were calculated independently in each epoch. The epoch was forward and backward mirrored to avoid edge artifacts, and wavelet convolution was applied for 100 logarithmically spaced frequencies between 2 and 80 Hz (Cohen, 2014). The resulting filtered complex time series was converted into a power time series, the mirrored copies discarded, and its mean was taken across the epoch. After repeating this procedure for all epochs, the mean was taken across epochs. This yielded a 32 (electrodes) X 100 (frequencies) matrix of power values. For more detail, see function getPower.m.

1/f trend slope:

The 1/f trend slope was calculated for each channel independently using the power spectra calculated above. Calculations followed the procedure outlined in (Donoghue et al., 2020). Calculations were carried out on the raw power spectra using the FOOOF algorithm in python. For more detail, see function fooofanalysis.py.

Peak alpha frequency:

Peak alpha frequency was calculated for each channel independently using the power spectra calculated above. Calculations followed the procedure outlined in Dickinson et al. (Dickinson, DiStefano, Senturk, & Jeste, 2018). Calculations were carried out on both the log-transformed and relative power spectra. Power values for frequencies between 6 and 14 Hz were used for calculation. These power values were detrended by subtracting the fitted 1/f trend line. Next, a gaussian curve was fit to the detrended power spectrum. The mean of this gaussian was taken as the peak alpha frequency. If the model fitting procedure failed to converge or if the mean of the gaussian fell outside the range 6-14 Hz, then this was taken as evidence that the participant did not have a strong alpha peak at that electrode and this participant's data were not interrogated further with respect to peak alpha frequency at that electrode. Regional means and asymmetry calculations (see below) ignored missing peak alpha frequencies at individual electrodes. 262/776 participants had at least one regional or asymmetry calculation that was not possible due to missing values for all electrodes involved in the calculation. However, no participant was missing more than 18 of 36 regional/asymmetry values. Only 22 participants were missing more than 5 values. In all, 97.7% of all peak alpha dependent measures were successfully fit. For more details, see function getSlopeAlpha.m

Phase-Amplitude Coupling:

Phase-amplitude coupling (PAC) was calculated independently in each epoch and for each channel. Calculations generally followed suggestions in (Peck et al., 2022; Tort, Komorowski, Eichenbaum, & Kopell, 2010). To do this, 10 low frequencies were chosen (2:20 Hz with spacing of 2 Hz). These frequencies were associated with linearly spaced standard deviation values of 2 to 3.5. 21 high frequencies were chosen (20:100 Hz with spacing of 4 Hz). These frequencies were associated with linearly spaced standard deviation values of 3.5 to 6. For each combination of low and high frequency, data were filtered using a frequency domain, gaussian-shaped filter convolution method, equivalent to time-domain wavelet convolution (Cohen, 2014). The low frequency complex time series was converted into a phase time series using the matlab angle function. The high frequency complex time series was converted into an amplitude time series using the matlab abs function. The high frequency amplitude time series

was divided into bins on the basis of the low-frequency phase time series. Specifically, the phase angles were split into 18 evenly-sized bins (i.e. 20 degrees per bin). The average high frequency amplitude was calculated for each bin. These mean values were normalized by the sum of the means across all bins.

For comparison, the same PAC calculation was carried out on data where the low frequency phase time series and the high frequency amplitude time series had been randomly temporally shifted relative to one another. In this way, it was possible to calculate the distribution of PAC strength that would be expected by chance. Temporal shifting and recalculation was carried out 200 times to build up a null distribution.

This procedure yielded an epoch X 10 (low frequency) X 21 (high frequency) X 18 (phase bin) X 32 (channel) matrix of observed PAC values and an associated 200 (random temporal shift repeats) X epoch X 10 (low frequency) X 21 (high frequency) X 18 (phase bin) X 32 (channel) matrix of null PAC values. The mean of both matrices was taken across the epoch dimension. Then, the Kullback-Leibler divergence was computed on both matrices across the phase bin dimension relative to a uniform distribution. This yielded matrices of epoch-averaged modulation indices for all combinations of channel, low frequency, and high frequency.

Observed modulation indices were converted into z-scores relative to their corresponding temporally shuffled null distributions.

Phase preference was calculated as the weighted circular mean of the bin phases, weighted by the mean amplitude values observed in each bin phase. Phase preference was calculated after averaging over epochs.

For more detail on how PAC values were calculated see function getPAC.m.

Multi-Scale Entropy:

Multi-Scale sample entropy (MSE) is a method that calculates sample entropy on both the original signal and coarse-grained time series derived from it. The algorithm consists of two steps that are performed independently for each electrode and epoch. First, the original signal is resampled to 1000Hz to ensure consistency across datasets. Then, coarse-grained time series are generated by averaging consecutive data points over time scales that increase with scale factor, resulting in a time series of length exactly divisible by the scale factor.(Busa & van Emmerik, 2016) Second, sample entropy is computed for each coarse-grained time series using a similarity threshold of 30% of the standard deviation of the time series denoted as γ, and a pattern length of m.(Costa, Goldberger, & Peng, 2005) Before computing sample entropy, all time series are centered and normalized to standard deviation 1 to avoid bias from amplitude variations. The distance between pairwise elements is calculated using the Chebyshev distance metric and the number of thresholded pairwise distances is counted.
and sample entropy equation is:

$$Sample\ Entropy(m,\ \gamma,\ N) \ = \ -\frac{N-m+1}{N-m-1} * log(\frac{A(m,\tau)}{B(m+1,\tau)})$$

where m is the pattern length (fixed at 2), τ is the time scale factor (from 1 to 20 in this study), N is the length of the original signal, $A(m,\ \tau)$is the number of pairs of vectors with a Chebyshev distance less than or equal to the similarity threshold for scale factor τ and pattern length m, and

$B(m + 1, \tau)$ is the number of pairs of vectors with a Chebyshev distance less than or equal to the similarity threshold for scale factor τ and pattern length $m + 1$.

This yielded an epochs X 20 (time scales) X 32 (channels) matrix. The mean of both matrices was taken across the epoch dimension. For more detail on how MSE values were calculated see functions getEntropyVals.m, Multi.m, and SampleEntropy.m.

Inter-Site Phase Clustering:

Inter-site phase clustering (ISPC) was calculated independently for every epoch between all electrode pairs within the same 100 logarithmically spaced frequencies between 2 and 80 Hz that were used for power spectrum calculation. Importantly, data were transformed using the surface Laplacian (custom matlab function laplacian_perrinX.m) prior to ISPC calculation. Calculation generally followed the procedure described in (Cohen, 2014). Specifically, for each frequency, data were first filtered using the same procedure described above. Next, filtered data were converted into a phase time series. Then, all pairwise channel comparisons were made using the following formula:

$$ISPC = \left| n^{-1} \sum_{t=1}^{n} e^{i(\phi_{xt} - \phi_{yt})} \right|$$

Here, n is the number of time points in the epoch, *i* is $\sqrt{-1}$, ϕ_{xt} is the phase angle of the signal from channel *x* at time *t*, and ϕ_{yt} is the corresponding value for channel y. This yielded an epochs X 100 (frequencies) X 32 (channels) X 32 (channels) matrix. Finally, the mean was taken with respect to epoch. For more detail on how ISPC was calculated see function getISPC.m.

Channel groupings for comparisons

All calculations generated values for every data channel (except ISPC, which generated values for pairs of channels), and for a very granular level of frequency resolution. However, signals at adjacent electrodes and adjacent frequencies are correlated. In addition, utilising all calculated values as dependent variables would be intractable (e.g. 32 electrodes X 100 frequencies = 3200 raw power variables alone). Thus, the preregistration plan was augmented by averaging variables into regional groups and canonical frequency bands.

The following 13 regional groupings were used: right frontal (FP2, AF4, F4, F8), left frontal (FP1, AF3, F3, F7), right centroparietal (FC2, FC6, C4, CP2, CP6), left centroparietal (FC1, FC5, C3, CP1, CP5), right occipito parietal (P4, P8, PO4, O2), left occipito parietal (P3, P7, PO3, O1), frontal (FP1, FP2, AF3, AF4, F4, FZ, F3), occipital (PO4, PO3, O2, OZ, O1), central (FZ, CZ, PZ, OZ), left lateral (F7, FC5, T7, CP5, P7), right lateral (F8, FC6, T8, CP6, P8), right hemisphere (FP2, AF4, F4, F8, FC6, FC2, T8, C4, CP6, CP2, P8, P4, PO4, O2), left hemisphere (FP1, AF3, F3, F7, FC5, FC1, T7, C3, CP5, CP1, P7, P3, PO3, O1).

Comparisons of particular asymmetries have also featured in the resting state autism literature.(Luschekina, Khaerdinova, Luschekin, & Strelets, 2017) (Carson, Salowitz, Scheidt, Dolan, & Van Hecke, 2014) Thus, we designed 5 asymmetry sensitive comparisons. In these comparisons, the second channel in each pair is being subtracted from the first, and the difference is divided by the sum of the two values (except for comparisons involving ISPC where the calculated values already represent pairs of electrodes). The mean of each set of subtractions was calculated. Specifically, we used the following sets: interhemispheric asymmetry: FP1-FP2, F3-F4, F7-F8, C3-C4, T7-T8, P3-P4, P7-P8, O1-O2; intrahemispheric asymmetry in the rostrocaudal direction in the left hemisphere: O1-P3, P3-C3, P7-T7, C3-F3, T7-F7, CP1-FC1; intrahemispheric asymmetry in the rostrocaudal direction in the right hemisphere: O2-P4, P4-C4, P8-T8, C4-F4, T8-F8, CP2-FC2; intrahemispheric asymmetry in the mediolateral direction in the left hemisphere: P7-P3, CP5-CP1, T7-C3, FC5-FC1, F7-F3; intrahemispheric asymmetry in the mediolateral direction in the right hemisphere: P8-P4, CP6-CP2, T8-C4, FC6-FC2, F8-F4. Note the importance of maintaining consistency in caudal, rostral and lateral, medial ordering across all pairs in these comparisons.

For power spectrum comparisons, spectra from each channel were averaged in 6 frequency bands: δ (2-4 Hz), θ (4-8 Hz), α (8-14 Hz), β (14-30 Hz), γlow (30-50 Hz), and γhigh (50-80 Hz). These averaged band power values were then used to calculate all regional and asymmetry comparisons. This process was repeated for raw, log-transformed, and relative power values. There were 324 (18 comparisons X 6 frequencies X 3 measures) dependent measures for power spectra.

For 1/f trend slope the regional and asymmetry comparisons were calculated for slope values derived from both log-transformed and relative power values. There were 36 (18 comparisons X 2 measures) dependent measures for 1/f trend slope.

For peak alpha frequency the regional and asymmetry comparisons were calculated for peak alpha frequencies derived from both log-transformed and relative power values. There were 36 (18 comparisons X 2 measures) dependent measures for peak alpha frequency.

For PAC, low frequencies were divided into δ (2-4 Hz), θ (4-8 Hz), α (8-14 Hz), β (14-20 Hz), and high frequencies were divided into β (20-32 Hz; note when β was compared to β, 24 Hz was used as the low cut off for the higher frequency), γlow (32-52 Hz), and γhigh (52-100 Hz). Thus, there were 12 pairs of frequency bands, and PAC was averaged within the range of each frequency band for every channel. Average PAC values were calculated for all regional and asymmetry comparisons using the PAC modulation index z-scored with respect to shuffle-generated null distributions. Note that the slight difference in frequency bands used here was done to replicate the bands used in a previous PAC study using resting state EEG data comparing AD to NT participants (Peck et al., 2022). In addition, it was important to have separation between the “low” and “high” frequencies since PAC fundamentally depends on comparison of signals between low and high frequencies. There were 216 (18 comparisons X 12 frequency pair) dependent measures for PAC.

For MSE, scale was divided into four ranges: all (1-20), fine (1-7), medium (8-13), coarse (14-20). MSE within each channel was averaged across these scale ranges. These averaged MSE values were used to calculate all regional and asymmetry comparisons. There were 72 (18 comparisons X 4 measures) dependent measures for MSE.

Finally, for ISPC, frequency was divided into the same 6 bands as above for the power spectrum. Within each band, the average ISPC was calculated for the electrode pairs specified in the 5 asymmetry comparisons (note: regional comparisons were not possible for ISPC as it requires pairs of electrodes). In addition, the mean long distance connectivity was calculated by taking the mean ISPC across all channel pairs whose inter-electrode euclidean distance was greater than the median inter-electrode euclidean distance. The mean short-distance connectivity was calculated similarly using pairs whose inter-electrode distance was less than the median. There were 42 (7 comparisons X 6 frequency bands) dependent measures for ISPC.

Thus, in total there were 728 (324 power + 36 1/f slope + 36 peak alpha + 216 PAC + 72 MSE + 42 ISPC) dependent measures.

Outlier removal

For some dependent variables, outliers were found to be so extreme that a small handful of values could drag the mean far above or below all other observations. In these cases, simply rejecting all observations more than some number of standard deviations from the mean would not work because all observations fell far from the mean. To get around this, the following two step procedure was adopted for each dependent variable. First, the mean and standard deviation of the middle 80% of the observed values were used to z-score all values. In this way, extreme outliers could not contribute to the standardisation. Second, all values with z-scores within 5 standard deviations were used to recalculate the mean and standard deviation for a final z-distribution. The data were then standardised according to this second set of mean and standard deviation values, and data points with z scores of greater than 5 in magnitude were deemed outliers and not included in the analysis. Outlier removal was done within the custom R function getModDat() in the helperFuncsFinal.R script.

Matched sample creation

Multicollinearity arises because of poorly matched samples wherein a variable of interest (i.e. diagnosis) varies with control variables (i.e. sex, age, IQ). A matched sample was created in which each AD and ASD participant was individually matched with a CON participant such that matched participants had the same sex, age within 5 months of each other, and IQ within 10 points of each other. Participants were drawn without replacement, and participants for whom a matched pair could not be found were discarded. All models were refit on this balanced subset of the data. Not all participants could be matched, so the final matched sample was smaller than the total dataset. The observed proportion of EEG variables that were associated with $\eta^2_{partial}$ above .035 was compared against the corresponding proportion derived from a bootstrap

analysis with non-matched samples of the same sample size. These values did not differ (Figure 4A), emphasising that there was no effect of multicollinearity in our results.

Extended sample size analysis

In order to assess changes in effect size as a function of sample size more completely, the entire data set (N=776) was considered as a whole without splitting it into age groups. In all other respects this analysis was the same as the analysis presented in the main text. Unsurprisingly, the number of variables for which a non-linear effect of age yielded a better fit was substantially higher than in the main analysis (54%) of variables. In this larger sample size, there were no EEG variables that could be predicted by diagnosis or any interaction involving diagnosis with $\eta^2_{partial}$ above .035. By contrast, there were 291 (40%) and 113 (16%) EEG variables that could be predicted by age and sex with $\eta^2_{partial}$ above .035, respectively. These values are comparable to the percentages obtained in the age group analysis, suggesting that a stable percentage of EEG variables can be predicted

Extended multicollinearity analysis

Multicollinearity is typically diagnosed by calculating the variance inflation factor (VIF) (Alin 2010). In the current analysis, we utilised the adjusted generalised standard error inflation factor (Fox & Monette, 1992), but we also pursued two additional checks.

First, two logistic regression models were fit using the glm function in R with family set to binomial:

Logistic M1: Diagnosis ~Age+Sex+IQ
Logistic M2: Diagnosis ~Age+Sex+IQ+EEG variable

The area under the receiver operating characteristic curve (AUC) associated with diagnosis classification was calculated for both models and the improvement of Logistic M2 relative to Logistic M1 was correlated with the corresponding ηpartial2 associated with Diagnosis in model M1. Multicollinearity affects coefficient estimates but does not affect a model's overall prediction ability. Thus, if multicollinearity had artificially suppressed ηpartial2 estimates in the main analysis, then AUC improvement should have been much larger than and unrelated to ηpartial2 estimates. This approach required that diagnosis be collapsed into a binary variable (AD/ASD vs. CON) for logistic regression.

The results of this analysis revealed that for no variable was AUC improved by more than 3%, indicating that there were no EEG variables capable of reliably predicting Diagnosis. There was a correlation between AUC improvement and ηpartial2 (r=.68; t(2182)=43.8; p<1*10e-15), indicating that the logistic regression approach yielded similar results to those of the multiple linear regression approach (supplemental Figure 2c).

Second, a related nested model approach was used with multiple linear regression to see if diagnosis could improve overall model fit:

Demographic Model: EEG dependent measure ~ Age*Sex+IQ+Age2
Residual Model: Demographic Model Residuals ~ Diagnosis

R2 of the Residual Model was evaluated as a measure of the power of diagnosis to predict variance in the EEG measures not contained in the demographic information. Again, because multicollinearity does not affect overall model prediction, this approach should have been less sensitive to multicollinearity than the main analysis. Correlation between R2 and ηpartial2 values was used to assess whether the Residual Model had revealed any unexpectedly strong predictions of diagnosis on the EEG data.

The results of this analysis revealed that for no variable was R2 greater than .065, indicating that there were no EEG variables that were well-predicted by Diagnosis. There was a correlation between R2 and ηpartial2 (r=.97; t(2182)=174.8; p<1*10e-15), indicating that the model comparison approach yielded similar results to those of the ηpartial2 based approach (supplemental Figure 2d).

High performance computing methods

Considering the large number of participants whose data contributed to this project and the comprehensive set of variables that were measured for each participant, the use of a high performance computing (HPC) cluster was necessary to complete this analysis within a manageable amount of time. Here, we present key considerations that facilitated the structuring of analysis code and formatting of data in order to use HPC resources effectively. The examples given throughout assume that the reader has downloaded the github repository and is able to view the scripts contained in it (https://github.com/adede1988/SheffieldAutismBiomarkers.git). It is our hope that the functions to compute specific aspects of EEG dynamics will prove useful to other researchers. The scripts and functions written to organize file paths and metadata, and that control loading data and saving outputs are all specific to the present analysis and are intended more as examples than as code that will be useful off the shelf to other researchers.

There are 4 key system considerations when switching from analysis on a local machine to using HPC resources. First, HPC is best suited to applications that require a high number of processors and a low amount of memory per processor. Second, management of HPC inputs and outputs requires a higher level of code and data organization than local analysis. Third, HPC resources are managed by scheduling software that will grant low priority to users who request large amounts of resources frequently. Fourth, because analysis using HPC clusters often involves processing a very large number of inputs, it encourages the user to automate many of the steps that are performed interactively during local analysis. These differences motivate how to structure analyses for optimal use of HPC resources.

1. *General code structure for interacting with HPC clusters*

When doing analysis on one local machine, users are accustomed to opening their preferred analysis software and then executing a series of code lines, function calls, or graphically selected menu options. This process is usually done for each subject and variable to be analyzed separately involving a high degree of customization and interaction throughout the analysis process. Often, the code associated with a project will end up being contained in one or a small handful of long scripts that require user edited file paths and parameter adjustments to work properly. This process can be very time consuming and difficult to keep track of. Analysis on an HPC encourages users to automate interactive steps, which will likely mean structuring code quite differently.

In addition to speeding up analysis, increasing automation when doing HPC analysis also improves memory usage and reproducibility. When analyzing one subject at a time on a local machine, users generally load entire datasets into memory and carry a large number of interim calculated variables through analysis. Although this can facilitate flexibility and interaction, it uses a lot of memory and it can be difficult to reproduce final results that are dependent on interim analysis steps. When analyzing using HPC clusters, it is best practice to load the minimum amount of data into memory at a time and to save interim outputs rather than to keep them loaded in memory.

For the present study, each script or function of analysis code could be categorized into one of four nested layers. Thinking of the code as belonging to these layers helps to understand the overall architecture of the code. Figure S1 displays a schematic representation of the interactions between the four code layers, the raw data, and the saved outputs. Specifically, Figure S1 displays initial multichannel analysis for one of the datasets we analyzed. To best understand this example, the relevant code files are available in the SheffieldAutismBiomarkers github repository. The example scripts are specific to the Autism Biomarkers Consortium for Clinical Trials dataset. As will be explained below, only layers 1 and 2 of the code were dataset specific. Layers 3 and 4 were dataset general. This structure allowed new datasets to be analyzed using the same computation code (layers 3 and 4) even if they required different formatting code (layers 1 and 2).

The defining feature of the code layers was that the code in the top layer was called from the command line on the HPC by the user. Code in each lower layer was called from the immediately higher layer. The top layer consisted of bash scripts, and we termed this layer the job initialization layer. These scripts described to the HPC cluster the computing resources required, the number of jobs to be done, the analysis software (Matlab in the present case) to be used for each analysis job, and the specific initializing commands to be sent to the Matlab command line to begin each job (see exampleBashScript.sh). One of these initializing commands was for Matlab to run one of the scripts belonging to the second layer.

When creating job initialization bash scripts, it is important to consider the system requirements of the particular HPC being used for analysis. The Sheffield Advanced Research Computer (ShARC) used for the present analysis relied on a job input and scheduling program called

SLURM (or simple linux utility for resource management). SLURM is a linux operating system scheduling program. It is commonly used on HPC systems. If implementing an HPC analysis on an HPC system that also uses SLURM, then it will be possible for users to use the same command structures shown in our exampleBashScript.sh. However, different HPC systems can use different scheduling and resource management systems, so it is important to check for compatibility as job initialization bash scripts may need to be adapted for the particular HPC that they are intended to run on. In most cases, research computing support staff are highly knowledgeable and helpful in properly formatting these initialization scripts.

The second layer consisted of pipeline wrapper scripts. The pipeline wrappers contained file paths to locate data and code and to specify where outputs should be saved. Depending on the application, they also specified the output format and populated metadata for the output. A key aspect of the wrappers was that they did not open any data files, and their main goals were formatting, job monitoring, and job initiation. They did no computation. The only truly necessary feature of any pipeline wrapper script was that it needed to call a pipeline function, the third code layer. The example script included in the github repository (getBioConsortDat.m) is written for the initial processing of the Autism Biomarkers Consortium for Clinical Trials dataset. It draws on a .csv file called biomarkConDat.csv to obtain metadata about participants. This file is also available in the github repository. The metadata were used to initialize the summary statistics file which contained interim output variables and was passed back and forth throughout the rest of the analysis (see green arrows in Figure S1).

The third layer consisted of pipeline functions. The pipeline loaded the raw data and then submitted the data to a series of computation steps to extract target variables. The pipeline kept output variables organized using the summary statistics file specified in the wrapper. Finally, the pipeline saved the summary statistics file (see setReadInAggregate.m).

A key difference between the pipeline wrapper and the pipeline itself was that the pipeline wrapper always included a directory of all relevant data files for the entire analysis, but it didn't actually open any data. By contrast, the pipeline dealt only with one data file path, and it did open those data. The main advantage of this separation was that multiple different wrappers could be combined with a single pipeline. This was critical to standardize analysis across different datasets. For example, note that the getBioConsortDat.m script is written for one specific dataset. Similar scripts were written for each dataset taking into account the specific idiosyncrasies of each dataset's file and data organization. No matter these differences, each wrapper script formatted file paths and metadata into the same format and then made a final call to the same pipeline (setReadInAggregate.m in this example). An additional advantage was that it was easy to change pipeline scripts while keeping the same wrapper(s), which facilitated analysis code development. Basically, the wrapper script answered the questions: which data are to be analyzed, where is the code to analyze them, and where should outputs be saved? The pipeline function then loaded the data, oversaw the analysis, and saved outputs.

Finally, the fourth code layer consisted of analysis computation functions. Each computation (cleaning, intersite phase clustering, power spectra, 1/f slope etc.) was contained in a separate

computation function. These computation functions were highly modular. Thus, it was easy to add or subtract computation functions from the analysis pipeline. Packaging computations into separate subfunctions also had the advantage of making the pipeline functions highly human readable. Most of the functions provided in the github repository are computation functions that extract measurements of particular features of the EEG. Each computation function took in data and summary statistics. They all saved summary statistics and processed data in addition to sending output back to the pipeline.

The result of these layers was that the code library for this analysis included many different code files, each with a highly specialized job.

2. *Development of HPC analysis code*

A few key concepts can help dramatically in developing analysis code using the multi-layer architecture used in the present study. Here, we describe four coding conventions that helped in the present project: file path control, debugging, modularity in code organization, and frequent output saving and progress checking. It is important to note that the code included in the github repository is intended primarily as an example that is specific to the present analysis. Implementing a similar analysis on a different HPC for a different analysis will require significant customization.

2.1 File path control

During development, code was written on a local machine and only deployed on the HPC cluster after completing debugging. Working locally during development facilitated easy interaction with the code and its outputs during development. The major challenge with this workflow is file path control.

Even when it is possible to utilize a network attached storage drive that can be viewed from both the HPC interface and one's local machine, the file paths used during HPC and local processing will be different. To get around this issue, file paths should be specified dynamically rather than statically. It is likely that in any scenario, it will only be the first part of any file path that will change between HPC and local processing. Thus, it is possible to specify the different part of all file paths as a single variable. For the present study, we used the convention that this variable was named "prefix". Note the variable named "prefix" specified at the top of getBioConsortDat.m. As a general rule, the prefix used for local processing should always be contained in a comment, and the prefix used for HPC processing should be in uncommented code. This will allow an interactive local user to run the local prefix but will prevent accidental file path incompatibility when moving to HPC analysis. Combining this convention with GitHub to keep code aligned between local machine and HPC cluster allowed for seamless movement between local and HPC analysis. In addition to using a prefix variable to standardize file paths, as a general rule, file paths should only be specified in job initiation and pipeline wrapper scripts. This rule makes file path errors easy to trace and fix.

2.2 Finding errors and debugging

Errors could occur during HPC processing for one of two general reasons. First, as with local processing, errors occurred because of mistakes in the analysis code. Second, errors could occur because of process failure on the HPC cluster.

Taking code errors first, it was important to be able to reproduce errors that occurred during analysis on the cluster on a local machine. It was much easier to examine the causes of errors when they could be reproduced on a local machine. To do this, errors had to be localized to the code layer (section 1) and particular script or function in which they occurred. Although there is no Matlab graphic user interface (GUI) when running analyses using an HPC cluster, the same error messages that would normally be displayed in the GUI are dumped into a text file in the current working directory on the cluster. Different HPC systems will have different conventions for the formatting of these text files, so it is important to check the specifics of your HPC system. A separate text file is created for each job run on the cluster. Thus, when initially moving from local code development to HPC analysis, it was important to run analyses on only a small subset of data in order to avoid generating an unwieldy number of error text files. In a similar fashion to the error messages, anything that would normally be displayed as text output in GUI-based interactive use of Matlab is dumped into an output text file when running code using an HPC cluster. Notice that throughout all four layers of code, frequent usage of the disp() function created outputs that were printed into the output text file. Combining the output text file and the error text file, it was possible to know how far the analysis had gotten before any error and exactly what error had caused it to stop.

Having identified the exact piece of code that caused the error, it was then possible to recreate the error on a local machine in GUI-based interactive mode. This was done by setting the jobID to the number displayed in the relevant text output file (to specify the data to be analyzed), setting the prefix for local processing, placing a debug stop point in the pipeline function prior to the point of the error, and then running the pipeline wrapper code to call the pipeline function. This procedure made it possible to examine and interact with the Matlab workspace at the time of the error, which had initially occurred on the cluster. The ability to reproduce errors that occurred during batch processing in an interactive Matlab session was critical to facilitating debugging.

Errors also occurred when jobs failed to finish or execute properly on the cluster. These errors often occurred when a job required more time or memory than had been allotted to it. These errors were not caused because of a problem with the code. Instead, the fix for these errors was often to rerun the analysis. However, for analysis steps with a very high job count, it was not efficient to rerun the entire analysis. It was also difficult if a large number of jobs had completed to know whether a small handful may not have completed properly. Thus, it was necessary to audit the analysis outputs.

The Matlab files, audit_singleChanAll.m and PACaudit.m work together to check whether phase amplitude coupling analysis has completed properly across a set of 88,160 individual EEG

channel files. As explained below, for the bulk of EEG metrics analyzed in this study, subject data files were split apart into individual channel data files. This facilitated parallel processing, but it also made it impossible to check all of the output and error text files associated with running an analysis on all channel data files. The auditing code specified in these two Matlab files checked through all 88,160 files and returned error codes organized as a 1-dimensional vector across the entire set of files, making it much easier for the user to assess progress and target particular data files for reprocessing or extra scrutiny. The audit code in these files took about 3 hours to run on a local machine capable of running 8 parallel processes, but if audit_singleChanAll.m and PACaudit.m are thought of as pipeline wrapper and pipeline functions respectively, then it would be simple to modify these for implementation on an HPC cluster through the addition of a job initiation bash script. Thus, one could audit the output of an HPC analysis using another HPC analysis if one's local machine is not powerful enough or the dataset in question is too large to run audit operations locally. Audit code was able to detect errors, but it was also useful for checking the progress of an analysis as it ran.

2.3 Modularity in coding

Coding modularly facilitates analysis development, makes errors easier to find, and increases the flexibility of code. To accomplish this, code writing was easiest to implement using a similar approach to debugging. For example, when writing a new computation function to perform a power spectrum decomposition, it was easiest to work on a local machine, set a debug stop point in the pipeline function at the point that the power spectrum decomposition was to be performed, and then initiate analysis from the pipeline wrapper. Once the code stopped at the debug point, it was then easy to see exactly what variables were available in the workspace and to implement code to perform the power spectrum decomposition given those variables. The new analysis code could be written in a typical trial and error fashion interactively. Then, once the analysis was working, it could be packaged into a new power spectrum decomposition function and separated into its own file separate from the pipeline function. In this way, it was easy to remove or add analyses to the pipeline. It was also easy to focus thinking on the development of only one piece of code at a time.

2.4 Frequent output saving and progress checking

When running large analyses on an HPC cluster, it was important to save interim results frequently and to check for previously completed computations. To do this, each computation function checked whether its output variables were already available before performing computation and only performed computation if the outputs were not already available. In addition, each computation function saved its outputs after completing computation (as indicated by the blue and green arrows pointing to the saved outputs from computation functions in Figure S1. In this way, work was not wasted even when the full pipeline failed to complete, a new computation function was added, or a computation function was edited. Without doing progress checks and saving outputs, it would not have been possible to complete this analysis in a reasonable amount of time.

3. Key steps of the present analysis

The present analysis was split into 7 functional steps: data harmonization, multi-channel analysis using HPC, splitting data into single channels, single-channel analysis using HPC, combining single channel results, combining subject results, inferential statistics. Figure S1 encompasses data harmonization, multi-channel analysis using HPC, and splitting data into single channels. Single-channel analysis using HPC followed a structure similar to that represented in Figure S1. The key differences were that rather than starting with raw data, the analysis began with the single-channel files that were output at the end of the analysis depicted in Figure S1. In addition, because all single-channel files were in a uniform format, there was no need for multiple pipeline wrapper functions for the different datasets. Below, we describe each of the analysis steps.

3.1 Data harmonization

To facilitate comparison of data collected across multiple sites, it was important to harmonize data. Harmonization also helped to streamline analysis since once data were formatted in a standard way, the same analysis code could be applied to all of the data. To harmonize the data, it was necessary to write a custom job initiation and pipeline wrapper script for each dataset. As explained above (section 1), these scripts requested resources, organized metadata, specified file paths, and called the pipeline script. The main reason for writing different scripts for each dataset was to deal with the different file structures in which their raw data were stored and the different locations where their metadata could be found. We chose to include only metadata that were available across all datasets in order to facilitate comparison.

The only other aspect of analysis that was customized for each dataset was the readEEGdat.m function, which included separate code blocks for loading raw data in different formats. All data were formated into a standard EEGLAB format (Delorme & Makeig, 2004). readEEGdat.m was called from within the multi-channel analysis pipeline function (setReadInAggregate.m)

3.2 Multi-channel HPC analysis

Although single channel analysis is more efficient for parallelization (section 4), certain analyses are fundamentally multi-channel. Thus, we implemented two rounds of HPC analysis. First, multi-channel analysis was done on data from whole subjects. Next, single-channel analysis was done on data broken down to the single channel level (sections 3.3 and 3.4).

Multi-channel analysis included the initial read in of the raw data, formatting the data, automated cleaning of the data, interpolation to a standard montage, connectivity analysis (intersite phase clustering), and splitting the data into single channels. Each of these operations was accomplished by a separate computation function within the setReadInAggregate.m pipeline function.

3.3 Splitting data into single channels

The final step of the setReadInAggregate.m pipeline function was to split the data into single channel files. The key things to consider when creating single channel files were creation of unique and systematically reproducible file names and sufficient metadata to make it easy to reaggregate all single channels associated with a given subject back together after analysis. In this analysis, we chose to store single channel files alongside the subject's raw data. Thus, the file directory of the raw data could be used to locate all of its associated channel files. This directory was stored in the summary data file associated with the subject, and each subject's summary data file then served as the core record for all analyses associated with that subject.

3.4 Single-channel HPC analysis

The bulk of EEG variables considered in the present study were computed on single channel data. These were accomplished through the construction of a single set of job initiation, pipeline wrapper, and pipeline code files to handle analysis for all channels. By using the same set of code for all channels regardless of which dataset they had originally come from, it was possible to be certain of uniformity in analysis methods.

Within the single channel pipeline, there were several computation functions. Specifically, we calculated power spectra, 1/f slope and intercept, alpha peak frequency, phase amplitude coupling, and multi-scale sample entropy.

3.5 Combining single channel results

Because the naming convention and save location of single channel files was determined by the information contained in the subject's summary data file, it was possible to load the summary data file and use its metadata to loop across the subject's associated single channel files. This allowed the subject level summary files to be updated with the results of the single channel analysis. Example code for performing this combination step can be seen in stitchFilesTogether.m.

3.6 Combining subject results

Considering the large number of variables extracted from each subject's data, we next sought to create standardized output variables that binned data across frequency and head topography. This was accomplished through the use of the extractingFinalVariables.m script. This script read in each subject's summary output data and then pulled a standard set of output variables. This step allowed data from all subjects to be represented in a single .csv file to facilitate inferential statistics.

Notice in both this and the previous section, the code used to implement the analysis step does not perform any actual computation or interact with the raw data in any way. As a result, these steps require much less computation time than the main analysis steps. Using the file path conventions discussed above, it was easy to write code for these steps that could run either

locally or on the HPC cluster. However, in practice, these steps could be run locally in a reasonable amount of time.

3.7 Inferential statistics

Finally, the .csv file generated in the combining subject results step was imported into R for final inferential statistical analysis. At this point, the .csv file could be imported into any environment for statistical analysis depending on the user's preference. Our inferential statistics were carried out using the script effectSizeCalculations.R.

4. Why splitting to single channels is so helpful on an HPC cluster

It is common practice to analyze data using a process that amounts to two nested loops. One loop is done across subjects. The second loop is done across data channels. This style of analysis does not take full advantage of the benefits of HPC analysis because of its poor compatibility with parallelization and its heavy memory usage. Take incompatibility with parallelization first, the power of HPC analysis comes from the ability to use many computing nodes simultaneously. If only one node is being asked to loop through all of the data, then there will be no speed gained from HPC use. For example, in the present analysis, we found that because of the bootstrapping required for calculating phase amplitude coupling, a single channel required approximately 1 hour of computing time. Thus, for a subject with 32 channels of data, analysis would take 1.5 days. For a typical study in which analysis is done as data are collected, this processing time may be acceptable. However, for the present study with 776 participants, this meant that approximately 3 years of compute time would have been needed to loop across all subjects and channels one at a time. This example suggests that simply parallelizing over subjects may solve the problem. If it were possible to process all subjects at the same time, then the entire analysis would only take 1.5 days to run, no matter how many subjects were in the study. However, this will run into problems with memory usage.

Typical desktop computers used for data analysis have 32 GB of memory, and in many cases they may have as much as 128 GB. Thus, when performing only one process at a time on a local machine, there is generally little need to worry about memory usage. However, when working with HPC clusters, there is often only 3-5 GB of memory per processor. The ShARC cluster used in the present study has 4 GB of memory per processor. This means that analyses which require more than 4 GB of memory will take memory resources away from other processors and be difficult for the HPC scheduling software to carry out while balancing demands from other users. In the case of using the ShARC, we found that when requesting less than 4 GB of memory per analysis job, it was possible to receive allocation to as many as 1000 simultaneous computer processors. By contrast, when requesting 64 GB of memory, it was rare to receive more than 4 computer processors. Thus, memory limitations are a primary reason why it is important to break out of both the subject and the channel loop when structuring analysis.

Figure S1 Caption.

General outline of multichannel analysis using HPC cluster. Code was organized into four layers. The top layer, the job initialization bash script, was called from the HPC linux command line. It requested resources from the cluster, specified the number of data files to be processed, initiated one job on the cluster per data file, opened Matlab, and called the pipeline wrapper script with the jobID number as an input. The pipeline wrapper Matlab script handled file path control, organized metadata, and called the pipeline function. The pipeline function loaded the raw data into Matlab and then submitted the raw data to a series of processing steps. Each processing step was executed by a different Matlab computation function. These computation functions were modular and could be easily plugged in or removed from the analysis pipeline. Black arrows indicate the flow of code based calls from one script or function to another. Green lines indicate the flow of subject specific summary statistics files. Pink lines indicate the flow of raw data. Light blue lines indicate the flow of processed data. Dark blue lines indicate the flow of single channel data. Ellipses within the fourth layer of code indicate that an arbitrary number of additional computation functions could be inserted within the pipeline. Ellipses between the left and right sides of the flow chart indicate that an arbitrary number of separate subject files could be processed in parallel.

Figure S2 Caption.

Measures of modeling EEG using diagnosis, sex, and age. **a.** Histograms display the replication rate of the ability of age and sex variables (vertical axis of pannels) to predict EEG dependent variables with η^2partial > .06. Replication rate was obtained by bootstrapping random half splits of the data and asking what proportion of splits yielded η^2partial > .035 in both halves. Colors indicate different categories of EEG variables. Data are collapsed across age group for visualisation. Vertical dashed lines indicate the threshold of replication rate = .64. Notice that when the threshold for inclusion is raised to η^2partial > .06 the shape of the distribution is more skewed to the right than in main text Figure 2b. **b**. Scatter plots display the relationship between effect sizes for the prediction of EEG variables across different predictors. Different independent predictors are labeled along the x and y axes of the plots. Each dot represents the two effect sizes associated with the two independent predictors labeled on the axes for one EEG variable. Vertical and horizontal lines represent the η^2partial = .035 threshold. Notice that very few points are found in the top right quadrant of the plots, indicating that diagnosis and different interaction terms including diagnosis are able to predict different aspects of the EEG. **c.** The scatter plot displays the results of a logistic regression model with diagnosis ~ EEG variable versus the results of the analysis presented in the main text. Specifically, the improvement in the area under the receiver operating characteristic curve (AUC) was calculated for the model comparison between a model including age, sex and IQ in predicting diagnosis versus a model that added an EEG variable. This improvement in AUC is plotted on the y axis and represents the marginal relationship between diagnosis and a given EEG variable. The η^2partial value associated with diagnosis from the main analysis is plotted on the x axis. Colors represent different age groups. Notice that there is a strong correlation between these measures and that

no EEG variable increases the AUC by more than .03. **d.** The scatter plot displays the results of an analysis predicting the residuals of a base model using diagnosis versus the results of the analysis presented in the main text. Specifically, the residuals were extracted from a model using age, sex, IQ, and age^2 to predict an EEG variable. These residuals were modeled as a function of diagnosis, and the R^2 of this residual model are plotted on the y axis. The η^2partial value associated with diagnosis from the main analysis is plotted on the x axis. Colors represent different age groups. Notice that there is a strong correlation between these measures.

Figure S3 Caption.

The variables best predicted by age replicate known findings from prior research. All scatter plots display individual participants. IQ is represented by the size of the points. Diagnosis is represented by the color of the points. Sex is represented by the shape of the points. Vertical dashed lines indicate the age group cut offs. Each plot displays results for the EEG variable best predicted by age within one age group. Although variables were detected in data from a single age group, all participants are plotted in each plot. **a.** Multi-scale entropy in the central scalp (y axis) increased with age (x axis). **b.** Log transformed theta power in the lateral aspect of the left hemisphere (y axis) decreased with age (x axis). **c.** Log transformed delta power across the right hemisphere (y axis) decreased with age (x axis). Note that many variables changed reliably with age. These are simply the three for which the effect size was greatest.

Bibliography

Busa, M. A., & van Emmerik, R. E. A. (2016). Multiscale entropy: A tool for understanding the complexity of postural control. *Journal of sport and health science*, *5*(1), 44–51.

Carson, A. M., Salowitz, N. M. G., Scheidt, R. A., Dolan, B. K., & Van Hecke, A. V. (2014). Electroencephalogram coherence in children with and without autism spectrum disorders: decreased interhemispheric connectivity in autism. *Autism research : official journal of the International Society for Autism Research*, *7*(3), 334–343.

Cohen, M. X. (2014). *Analyzing neural time series data: theory and practice*. The MIT Press.

Costa, M., Goldberger, A. L., & Peng, C. K. (2005). Multiscale entropy analysis of biological signals. *Physical Review E*, *71*(2).

Dede, A. (2023). Investigating possible biomarkers of autism in resting EEG. *NIMH Data Repositories*.

Delorme, A., & Makeig, S. (2004). EEGLAB: an open source toolbox for analysis of single-trial EEG dynamics including independent component analysis. *Journal of Neuroscience Methods*, *134*(1), 9–21.

Dickinson, A., DiStefano, C., Senturk, D., & Jeste, S. S. (2018). Peak alpha frequency is a neural marker of cognitive function across the autism spectrum. *The European Journal of Neuroscience*, *47*(6), 643–651.

Donoghue, T., Haller, M., Peterson, E. J., Varma, P., Sebastian, P., Gao, R., Noto, T., et al. (2020). Parameterizing neural power spectra into periodic and aperiodic components. *Nature Neuroscience*, *23*(12), 1655–1665.

Fox, J., & Monette, G. (1992). Generalized Collinearity Diagnostics. *Journal of the American Statistical Association*, *87*(417), 178–183.

Hyatt, C. J., Wexler, B. E., Pittman, B., Nicholson, A., Pearlson, G. D., Corbera, S., Bell, M. D., et al. (2022). Atypical Dynamic Functional Network Connectivity State Engagement during Social-Emotional Processing in Schizophrenia and Autism. *Cerebral Cortex*, *32*(16), 3406–3422.

Lord, C., Rutter, M., DiLavore, P., Risi, S., Gotham, K., & Bishop, S. (2012). *Autism diagnostic observation schedule--2nd edition (ADOS-2)*. Los Angeles, CA: Western Psychological Corporation.

Luschekina, E. A., Khaerdinova, O. Yu., Luschekin, V. S., & Strelets, V. B. (2017). Interhemispheric differences in the spectral power and coherence of EEG rhythms in children with autism spectrum disorders. *Human physiology*, *43*(3), 265–273.

Marin, A., Hutman, T., Ponting, C., McDonald, N. M., Carver, L., Baker, E., Daniel, M., et al. (2020). Electrophysiological signatures of visual statistical learning in 3-month-old infants at familial and low risk for autism spectrum disorder. *Developmental Psychobiology*, *62*(6), 858–870.

McPartland, J. C., Bernier, R. A., Jeste, S. S., Dawson, G., Nelson, C. A., Chawarska, K., Earl,

R., et al. (2020). The Autism Biomarkers Consortium for Clinical Trials (ABC-CT): Scientific Context, Study Design, and Progress Toward Biomarker Qualification. *Frontiers in Integrative Neuroscience*, *14*, 16.

Neuhaus, E., Lowry, S. J., Santhosh, M., Kresse, A., Edwards, L. A., Keller, J., Libsack, E. J., et al. (2021). Resting state EEG in youth with ASD: age, sex, and relation to phenotype. *Journal of neurodevelopmental disorders*, *13*(1), 33.

Ozonoff, S., Young, G. S., Bradshaw, J., Charman, T., Chawarska, K., Iverson, J. M., Klaiman, C., et al. (2024). Familial recurrence of autism: updates from the baby siblings research consortium. *Pediatrics*, *154*(2).

Peck, F., Naples, A. J., Webb, S. J., Bernier, R. A., Chawarska, K., Dawson, G., Faja, S., et al. (2022). Phase-Amplitude Coupling in Autism Spectrum Disorder: Results from the Autism Biomarkers Consortium for Clinical Trials. *medRxiv*.

Tamminga, C. A., Pearlson, G., Keshavan, M., Sweeney, J., Clementz, B., & Thaker, G. (2014). Bipolar and schizophrenia network for intermediate phenotypes: outcomes across the psychosis continuum. *Schizophrenia Bulletin*, *40 Suppl 2*(Suppl 2), S131-7.

Tort, A. B. L., Komorowski, R., Eichenbaum, H., & Kopell, N. (2010). Measuring phase-amplitude coupling between neuronal oscillations of different frequencies. *Journal of Neurophysiology*, *104*(2), 1195–1210.

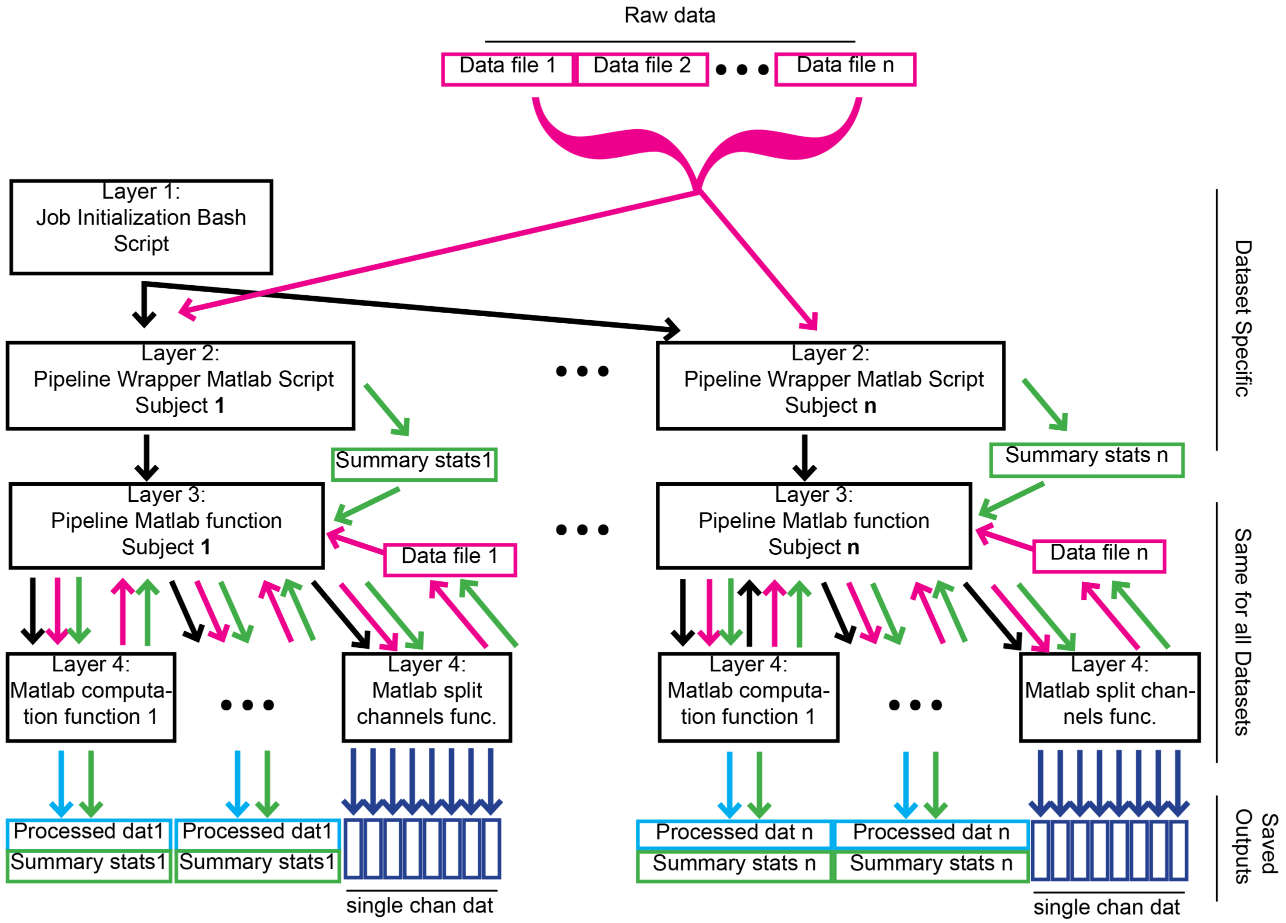

Supplemental Figure 1. General outline of multichannel analysis using HPC cluster. Code was organized into four layers. The top layer, the job initialization bash script, was called from the HPC linux command line. It requested resources from the cluster, specified the number of data files to be processed, initiated one job on the cluster per data file, opened Matlab, and called the pipeline wrapper script with the jobID number as an input. The pipeline wrapper Matlab script handled file path control, organized metadata, and called the pipeline function. The pipeline function loaded the raw data into Matlab and then submitted the raw data to a series of processing steps. Each processing step was executed by a different Matlab computation function. These computation functions were modular and could be easily plugged in or removed from the analysis pipeline. Black arrows indicate the flow of code based calls from one script or function to another. Green lines indicate the flow of subject specific summary statistics files. Pink lines indicate the flow of raw data. Light blue lines indicate the flow of processed data. Dark blue lines indicate the flow of single channel data. Ellipses within the fourth layer of code indicate that an arbitrary number of additional computation functions could be inserted within the pipeline. Ellipses between the left and right sides of the flow chart indicate that an arbitrary number of separate subject files could be processed in parallel.

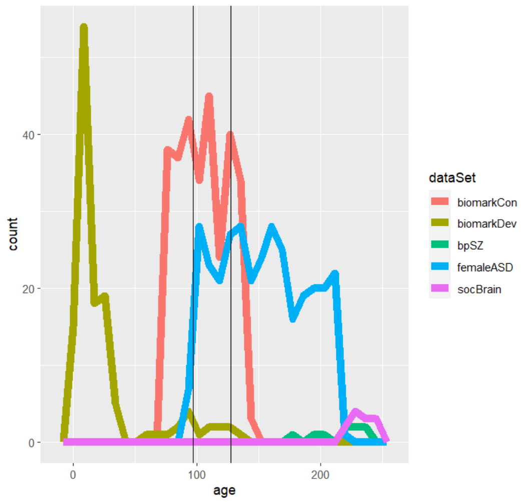

Supplemental Figure 2. Distribution of participant ages from different original datasets. X axis displays age in months. Y axis displays participant counts at different ages. Vertical black lines indicate boundaries between age groups. Age group boundaries were set such that each age group had an equal number of participants. Note that although different original datasets contributed participants with different age distributions, all three of our main datasets contributed to all three age groups. In addition, many age effects spanned all three age groups smoothly (See Supplemental Figure 5 for three examples). Dataset names are as in Supplemental Table 1.

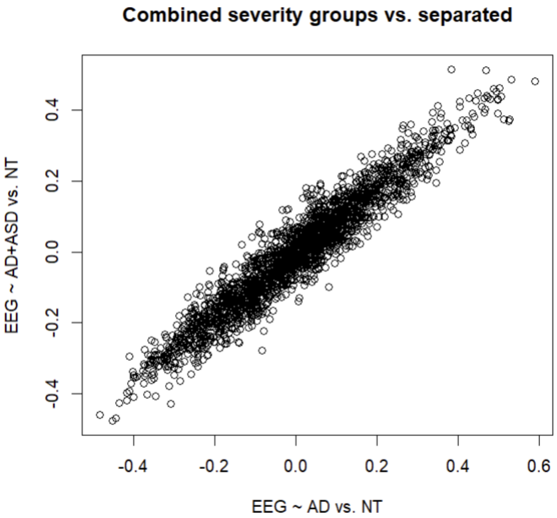

Supplemental Figure 3. In our main analysis, autistic participants were split into a relatively severe AD group and a relatively mild ASD group. We also performed our core analysis with AD and ASD groups combined. Here, the x-axis indicates the beta coefficients associated with the difference between AD and NT groups when there was a separate ASD group. The y-axis indicates the corresponding beta coefficients associated with the difference between AD+ASD participants combined into a single group and the NT group. There is a strong correlation between these measures (r=.96; p<2e10^-16), indicating that the severity split did not make a major impact on the interpretation of differences between AD and NT groups.

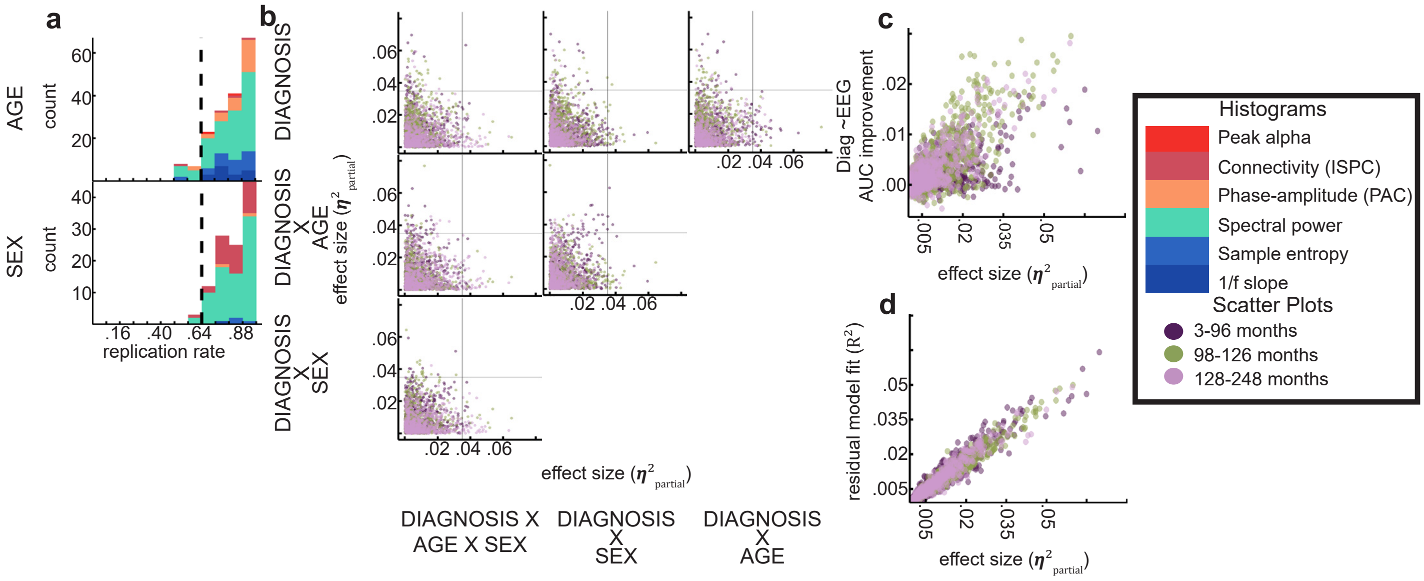

Supplemental Figure 4. Measures of modeling EEG using diagnosis, sex, and age. a. Histograms display the replication rate of the ability of age and sex variables (vertical axis of pannels) to predict EEG dependent variables with $\eta^2_{partial}$ > .06. Replication rate was obtained by bootstrapping random half splits of the data and asking what proportion of splits yielded $\eta^2_{partial}$ > .035 in both halves. Colors indicate different categories of EEG variables. Data are collapsed across age group for visualisation. Vertical dashed lines indicate the threshold of replication rate = .64. Notice that when the threshold for inclusion is raised to $\eta^2_{partial}$ > .06 the shape of the distribution is more skewed to the right than in main text Figure 2b. **b.** Scatter plots display the relationship between effect sizes for the prediction of EEG variables across different predictors. Different independent predictors are labeled along the x and y axes of the plots. Each dot represents the two effect sizes associated with the two independent predictors labeled on the axes for one EEG variable. Vertical and horizontal lines represent the $\eta^2_{partial}$ = .035 threshold. Notice that very few points are found in the top right quadrant of the plots, indicating that diagnosis and different interaction terms including diagnosis are able to predict different aspects of the EEG. **c.** The scatter plot displays the results of a logistic regression model with diagnosis ~ EEG variable versus the results of the analysis presented in the main text. Specifically, the improvement in the area under the receiver operating characteristic curve (AUC) was calculated for the model comparison between a model including age, sex and IQ in predicting diagnosis versus a model that added an EEG variable. This improvement in AUC is plotted on the y axis and represents the marginal relationship between diagnosis and a given EEG variable. The $\eta^2_{partial}$ value associated with diagnosis from the main analysis is plotted on the x axis. Colors represent different age groups. Notice that there is a strong correlation between these measures and that no EEG variable increases the AUC by more than .03. **d.** The scatter plot displays the results of an analysis predicting the residuals of a base model using diagnosis versus the results of the analysis presented in the main text. Specifically, the residuals were extracted from a model using age, sex, IQ, and age^2 to predict an EEG variable. These residuals were modeled as a function of diagnosis, and the R^2 of this residual model are plotted on the y axis. The $\eta^2_{partial}$ value associated with diagnosis from the main analysis is plotted on the x axis. Colors represent different age groups. Notice that there is a strong correlation between these measures.

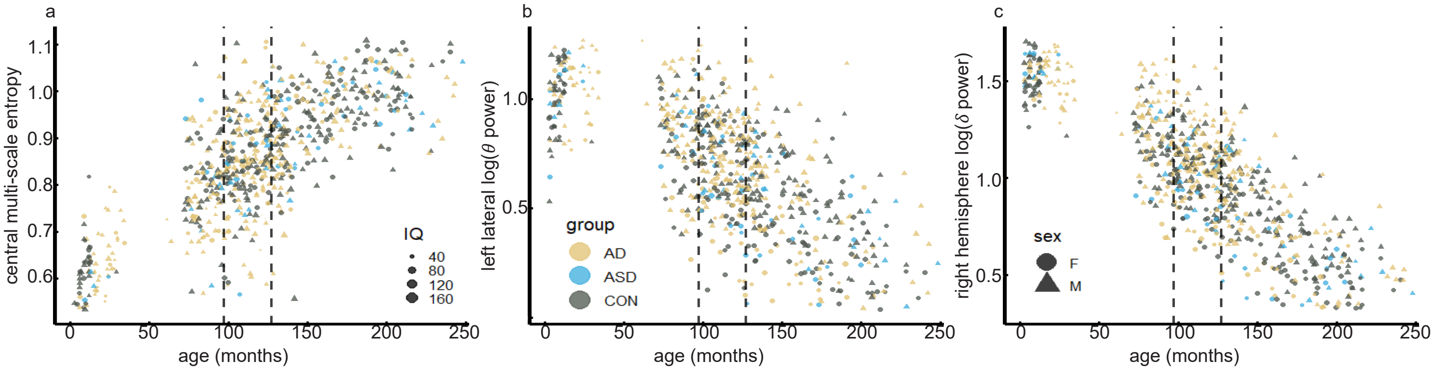

Supplemental Figure 5. The variables best predicted by age replicate known findings from prior research. All scatter plots display individual participants. IQ is represented by the size of the points. Diagnosis is represented by the color of the points. Sex is represented by the shape of the points. Vertical dashed lines indicate the age group cut offs. Each plot displays results for the EEG variable best predicted by age within one age group. Although variables were detected in data from a single age group, all participants are plotted in each plot. **a.** Multi-scale entropy in the central scalp (y axis) increased with age (x axis). **b.** Log transformed theta power in the lateral aspect of the left hemisphere (y axis) decreased with age (x axis). **c.** Log transformed delta power across the right hemisphere (y axis) decreased with age (x axis). Note that many variables changed reliably with age. These are simply the three for which the effect size was greatest.

Data Set	Key reference	Paradigm	ASD Participants	Neurotypical Participants	collection ID
biomarkCon	McPartland et al., 2020	Viewing of abstract videos	Screened with ADOS and ADI-R	Exclusionary criteria: neurological disorder/serious head injury, use of benzodiazepines, barbiturates, carbamazepine, and/or valproic acid, presence of sensory/motor impairment, IQ <70	2288
biomarkDev	Ozonoff et al., 2024* Marin et al., 2020	Viewing videos of bubbles	Babies recruited based on familial risk of autism (older diagnosed siblings). Diagnosed with ADOS and ADI-R at ~36 months of age.	Exclusionary criteria: neurological abnormalities, birth-related complications, uncorrected vision or hearing impairment, or first degree relative with ASD	2026
femaleASD	Neuhaus et al., 2021	Dynamic screensaver type videos	Screened with ADOS and ADI-R	Screened via clinician impression, SRS-2 and SCQ	2021
socBrain	Hyatt et al., 2022^	eyes open rest	Screened with ADOS	Screened with ADOS	2022
bpSZ	Tamminga et al., 2014	eyes open rest	N/A	Healthy volunteers without psychotic illness, or in their immediate family.	2274

Supplementary Table 1

ADOS = Autism Diagnostic Observational Schedule

ADI-R = Autism Diagnostic Inventory - Revised

SRS = Social Responsiveness Scale

SCQ = Social Communication Questionnaire

biomarkCon = The Autism Biomarkers Consortium for Clinical Trials

biomarkDev = Biomarkers of Developmental Trajectories and Treatment in ASD

bpSZ = Bipolar & Schizophrenia Consortium for Parsing Intermediate Phenotypes

femaleASD = Multimodal Developmental Neurogenetics of Females with ASD

socBrain = The Social Brain in Schizophrenia and Autism Spectrum Disorders

*only a subset of the participants in this study had EEG data and were either controls or diagnosed with autism.

^This publication reflects the participant recruitment methods used to obtain the data used in the present analysis. However, this publication presents fMRI data and not the EEG data analyzed in the present study. Records on the NIMH data Archive do not clearly indicate where results based on the EEG data analyzed here may have been published previously.
